# Synthesis and Structure-Activity Relationships of Imidazole-Coumarin Conjugates against Hepatitis C Virus

**DOI:** 10.3390/molecules21020228

**Published:** 2016-02-18

**Authors:** Shwu-Chen Tsay, Shu-Yu Lin, Wen-Chieh Huang, Ming-Hua Hsu, Kuo Chu Hwang, Chun-Cheng Lin, Jia-Cherng Horng, I-Chia Chen, Jih Ru Hwu, Fa-Kuen Shieh, Pieter Leyssen, Johan Neyts

**Affiliations:** 1 Department of Chemistry & Frontier Research Center on Fundamental and Applied Sciences of Matters, National Tsing Hua University, Hsinchu 30013, Taiwan; kchlsu@mail2000.com.tw (S.-Y.L.); jordanback2001@yahoo.com.tw (W.-C.H.); minghua.hsu@gmail.com (M.-H.H.); kchwang@mx.nthu.edu.tw (K.C.H.); cclin66@mx.nthu.edu.tw (C.-C.L.); jchorng@mx.nthu.edu.tw (J.-C.H.); icchen@mx.nthu.edu.tw (I.-C.H.); 2 Department of Chemistry, National Central University, Jhongli District, Taoyuan City 32001, Taiwan; fshieh@ncu.edu.tw; 3 Rega Institute for Medical Research, Katholieke Universiteit Leuven, Minderbroedersstraat 10, Leuven B-3000, Belgium; pieter.leyssen@rega.kuleuven.be

**Keywords:** imidazole, nitrogen heterocycles, coumarin, hepatitis C virus, structure–activity relationships

## Abstract

A series of new conjugated compounds with a –SCH_2_– linkage were synthesized by chemical methods from imidazole and coumarin derivatives. The experimental results indicate that of the twenty newly synthesized imidazole–coumarin conjugates, three of them exhibited appealing EC_50_ values (5.1–8.4 μM) and selective indices >20 against hepatitis C virus. Their potency and selectivity were increased substantially by modification of their structure with two factors: imidazole nucleus with a hydrogen atom at the N(1) position and coumarin nucleus with a substituent, such as Cl, F, Br, Me, and OMe. These guidelines provide valuable information for further development of conjugated compounds as anti-viral agents.

## 1. Introduction

Hepatitis C virus (HCV) infection afflicts ~150 million people worldwide (~3% of the global population), with approximately 3–4 million new cases occurring annually [1]. HCV generally causes both mild and acute liver disease, possibly leading to cirrhosis, hepatocellular carcinoma, and liver failure. The traditional therapeutic treatment involves administering interferon α-2 or its PEGylated form, either alone or in combination with ribavirin [2,3]. In 2011, boceprevir and telaprevir were approved for the treatment of chronic hepatitis C genotype 1 infection in combination with peg-interferon α and ribavirin. The recommended treatment regimens have significantly increased the overall cure rates [4,5]. The above therapeutic treatments, however, still have substantial adverse effects [6]. During the past two years, the U.S. Food and Drug Administration has approved Harvoni™ (ledipasvir/sofosbuvir), simeprevir, sofosbuvir, and Viekira Pak™ (ombitasvir/paritaprevir/ritonavir tablet; dasabuvir tablet). Harvoni™ and Viekira Pak™ are combination pills in which each of the active ingredients therein has a distinct mechanism of action (MOA) [7,8]. As new generation drugs for the treatment of HCV infections, the recommended 12- or 24-week courses of therapy of both are highly expensive [9]. Thus, pharmaceutical companies are still seeking chemical entities with safer and lower price than the existing drugs to combat the HCV disease. Our recent works introduced a series of coumarin-containing conjugated compounds as new anti-HCV agents, including benzimidazole–, heterobicycle–, and (ribosyl)purine–coumarin conjugates [10,11,12,13,14]. The aim of this work was to synthesize new compounds with a scaffold containing a coumarin moiety that is conjugated with an imidazole moiety or its derivatives, including (1-ribofuranosyl)imidazole, inosine, and guanosine. Their anti-HCV activity is to be explored and their structure–activity is to be deduced.

Coumarins belong to an important family of compounds with various pharmacological functions [15,16]. Despite the development of their derivatives as HCV NS3·4A protease inhibitors and NS5B polymerase inhibitors [15,17], the flat and shallow substrate-binding groove of proteases inhibits their potency and selectivity [18,19].

Imidazole derivatives and imidazole-2-thiones represent a class of compounds with important pharmacological properties, including anti-bacterial [20], anti-inflammatory [21], anti-cancer [22], and anti-viral activities [23,24]. In the histidine moieties of enzymes, the imidazole ring functions as a proton donor or acceptor or both in enzymic reactions in the charge-relay system when it is in a free base form [25]. Therefore, many well-known biocatalysts possess this moiety. Several investigations have elucidated the *N*-alkylation of imidazole compounds, which alter the pharmacological actions and pharmacokinetics [26,27].

In 2010, Zai and co-workers [28] reported that inosine alters gene expression and axonal projections in neurons contralateral to a cortical infarct. Additionally, it exerts a broad range of anti-inflammatory effects in a murine model of acute lung injury, as found by Liaud *et al.* [29]. Inosine is also the major component of the drug isoprinosine for the treatment of chronic hepatitis B. This drug inhibits replication of many RNA and DNA viruses *in vivo* and *in vitro* [30]. It also exerts an immunostimulatory effect by enhancing T-cell function and macrophage activity [31]. Containing inosine as a major component, the drug Isoprinosine unfortunately exhibits several side effects [32]. Those effects include dyspepsia, hypersensitivity reactions, and severe drug reactions with ribavirin that may result in a drop in the white blood cell count of patients.

Giuliani and co-workers [33] recognized protective activity of guanosine in an *in vitro* model of Parkinson’s disease. Very recently, Gosselin *et al.* [34] reported a 2′-C-methyl branched guanosine pro-nucleotide as a potent liver-targeted HCV polymerase inhibitor. Schaefer–Korting *et al.* [35] found that a guanosine-analog phosphonate can improve topical non-melanoma skin cancer treatment.

**Figure 1:**
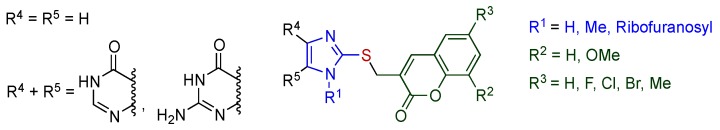
General structure of targed imidazole–coumarin conjugates.

This work extends the range of available benzimidazole–coumarin conjugates by replacement of the benzimidazole moiety therein with an imidazole moiety or its derivatives (*i.e.*, inosine and guanosine). An attempt is also made to understand how they influence the anti-HCV activity of the conjugated compounds by use of three structural relatives, namely *N*-H, *N*-methyl, and *N*-ribofuranosyl imidazoles. Additionally, various substituents (*i.e.*, F, Cl, Br, Me, and OMe) are attached to the coumarin moiety. In total, 20 imidazole–coumarin conjugated compounds with the common skeleton shown in Figure 1 below were synthesized and their structure–activity relationships derived.

## 2. Results and Discussion

### 2.1. Chemistry

To investigate the effects of different moieties or functional groups attached to the core, our research group synthesized four series of imidazole–coumarin conjugated compounds. The corresponding synthetic procedures are described as follows.

#### 2.1.1. Synthesis of Imidazole–Coumarin Conjugates

**Scheme 1:**
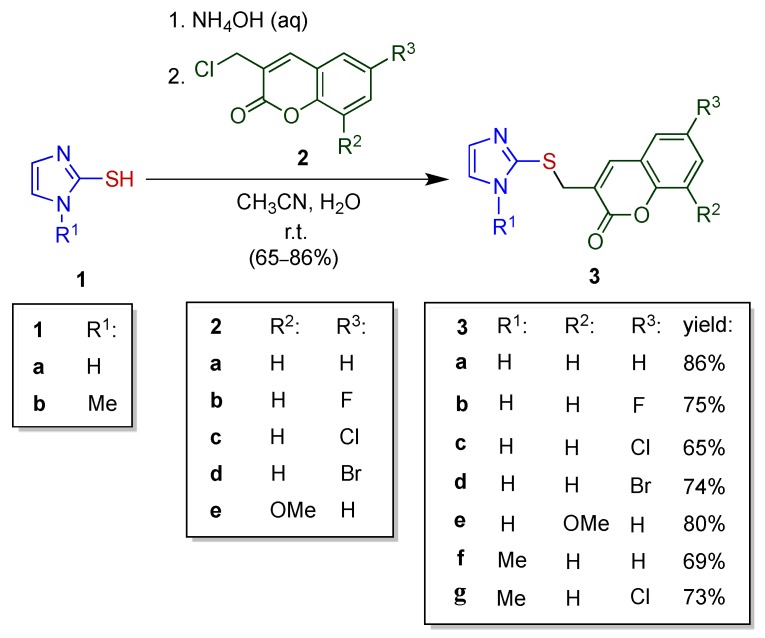
Synthesis of imidazole–coumarin conjugates.

Conjugates with a coumarin moiety attached at the C(2)-position of the imidazole group via a thiomethylene joint were synthesized. Correspondingly, 1*H*-imidazole-2-thiol (**1a**) was coupled with various 3-(chloromethyl)coumarins **2** in the presence of aqueous ammonia and acetonitrile. Conjugates **3a**–**e** with different substituents (e.g., F, Cl, Br, and OMe) were obtained in 65%–86% yields. Furthermore, their methyl analogs (*i.e.*, **3f** and **3g**) were prepared by alkylation of 1-methylimidazole-2-thiol (**1b**) with coumarins **2a** and **2c**, respectively. Under these alkaline conditions, the NH group of **1** did not compete with the C(2)-thiol group for nucleophilic attack of the coumarin substrates [12] (Scheme 1).

#### 2.1.2. Synthesis of (1-Ribofuranosyl)imidazole–Coumarin Conjugates

**Scheme 2:**
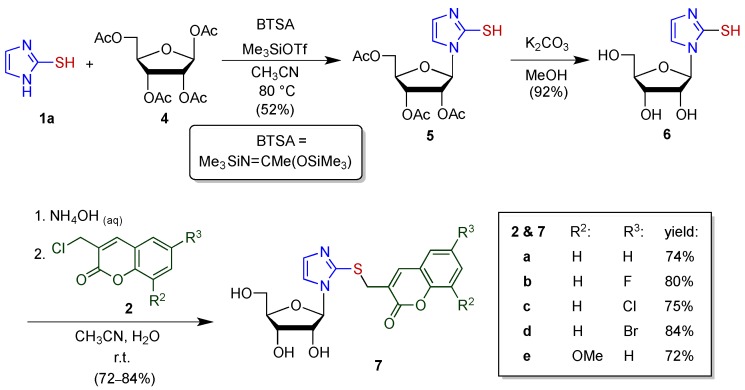
Synthesis of (1-ribofuranosyl)imidazole–coumarin conjugates.

Nucleoside derivatives are promising candidates for anti-viral drugs [36,37], which explains our intention to prepare (1-ribofuranosyl)imidazole–coumarin conjugates for an anti-viral activity assay. First, silylation of 1*H*-imidazole-2-thiol (**1a**) with *N*,*O*-bis(trimethylsilyl)acetamide (BTSA) [38] gave the intermediates, which were then coupled with 1,2,3,5-tetra-*O*-acetyl-β-d-ribofuranose (**4**) in the presence of Me_3_SiOTf and acetonitrile at 80 °C. Subsequent removal of the three acetyl groups in **5** with K_2_CO_3_ in methanol provided the resultant thiol **6**. Its coupling [39] with coumarins **2** produced (1-ribofuranosyl)imidazole–coumarin conjugates **7** in good yields (72%–84%) (Scheme 2).

#### 2.1.3. Synthesis of Inosine– and Guanosine–Coumarin Conjugates

**Scheme 3:**
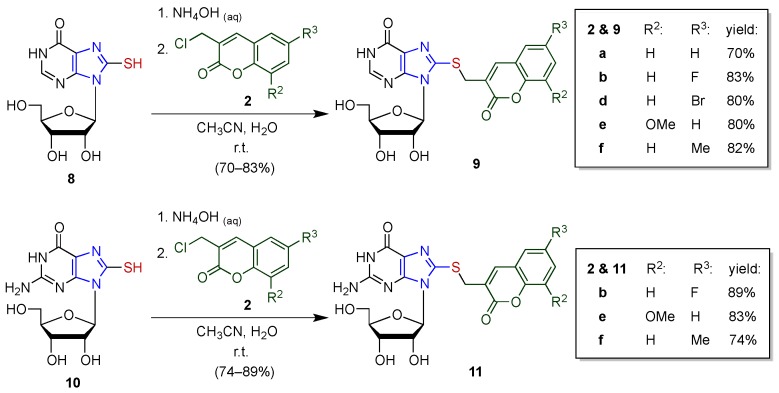
Synthesis of inosine– and guanosine-coumarin conjugates.

We found that the desired S_N_2 reactions occurred at the thiol group of compounds **8** [40] and **10** [41] with the allylic position of 3-(chloromethyl)coumarins **2** to give the desired conjugated products **9** and **11**, respectively. The unwanted Michael addition did not occur at the α,β-unsaturated lactone moiety of coumarins **2**. The key factors for success involved the use of 35% aqueous ammonia and acetonitrile as the media at room temperature. Accordingly, yields of 70%–83% for inosine–coumarins **9** and 74%–89% for guanosine–coumarins **11** were obtained (Scheme 3).

#### 2.1.4. Identification of the Structures of New Conjugated Compounds

The structures of all newly synthesized compounds were confirmed on the basis of their spectroscopic characteristics. For instance, the IR spectrum of conjugates **7a** displayed a strong absorption band at 1698 cm^−1^ , which was contributed to the carbonyl stretching vibration of the coumarin moiety [42]. Its ^13^C-NMR spectrum had resonances at 34.27 and 151.94 ppm for the SCH_2_ carbon and the –N=C(–N)(–S) carbons, respectively. Furthermore, a doublet with *J* = 7.2 Hz occurred at 5.67 ppm for the glycosidic proton [43] in its ^1^H-NMR spectrum. Nevertheless, it was beyond our expection that the two characteristic singlets, instead of doublets, occurred at 7.49 and 6.99 ppm for the protons in the imidazole nucleus. Our data are in consistent with those of *N*-(β-d-ribofuranosyl)imidazole, the parent compound of **7a**, as reported by Mourabit and co-workers [44].

In addition, a doublet with *J* = 6.4 Hz at 5.76 ppm in the ^1^H-NMR spectrum was assigned as the anomeric hydrogen of compound **9a**. They also exhibited two characteristic doublets at 4.39 and 4.34 ppm with *J* = 14.2 Hz for the two SCH_2_ hydrogens and a singlet at 8.06 ppm for the NH–CH=N proton in inosine moiety. In its ^13^C-NMR spectrum, resonance occurred at 32.04 ppm for the SCH_2_ carbon.

### 2.2. Pharmacology

#### Anti-HCV Activity

The antiviral activity of conjugated compounds **3a**–**g**, **7a**–**e**, **9a**,**b**,**d**–**f**, and **11b**,**e**,**f** in the HCV genotype 1b subgenomic Huh 5-2 replicon system [45] was evaluated according to established procedures [46]. On the basis of the dose-response curves that were obtained, the concentration of a compound that inhibited virus replication by 50% (*i.e.*, EC_50_) and the concentration of compound that reduced host cell metabolism by 50% (*i.e.*, CC_50_) were obtained. These values subsequently allowed us to calculate the selectivity index (*i.e.*, SI = CC_50_/EC_50_), which is a measure for the therapeutic window of the compound in an assay system. Compounds were only considered as selective inhibitors in the replicon assay when virus RNA replication was significantly inhibited (>70%) at concentrations not adversely affecting the host cell metabolism. The observed antiviral effect of other compounds was most likely related mainly to the pleiotropic or aspecific effect on the host cell.

Of the 20 newly synthesized conjugates, five (*i.e.*, **3b**–**f**) exhibited appealing antiviral activity with EC_50_ values of 5.1–9.7 μM in the HCV 1b Huh 5-2 replicon system (Table 1). Three conjugates **3b**, **3d**, and **3e** displayed a significant window of selectivity with SI of 12–21.

## 3. Structure–Activity Relationship: Essential Moieties and Functional Groups

This work established four new sets of conjugated compounds in the family of imidazole–, (1-ribofuranosyl)imidazole–, inosine–, and guanosine–coumarins as shown in Scheme 1, Scheme 2 and Scheme 3. All of the conjugates had a –SCH_2_– joint to connect a coumarin moiety to an imidazole moiety. The substituents in these conjugated compounds included F, Cl, Br, Me, and OMe. Close examination of their EC_50_, CC_50_, and SI values shown in Table 1 allows us to deduce their SAR.

Attachment of the coumarin moiety to the imidazole nucleus generated conjugates with important anti-HCV activity. Successful examples include **3b**–**e** with EC_50_ = 5.1–9.7 μM and SI = 8.8–21. Besides, attachment of a substituent (e.g., F, Cl, Br, and OMe) to the coumarin nucleus increased the potency and the SI value by a factor of 2.1–5.1 (*cf.* **3b**–**e** *vs.* **3a**).

An Me group could be introduced onto the imidazole nucleus, while the resultant conjugates without an N–H proton maintained a similar order of anti-HCV activity and selectivity (*cf.* **3f** and **3g** with EC_50_ = 6.7 and 15 μM, respectively; SI = 7.3 and 5.1, respectively). On the other hand, introduction of a β-d-ribofuranosyl moiety to the thiomethylene-linked imidazole–coumarin conjugates led to a triply hybrid compound (*i.e.*, **7a**–**e**), some of which also exhibited anti-HCV activity (EC_50_ = 14 and 19 μM for **7c** and **7d**, respectively). In contrast to the ribosylated form at the N(1) position of the imidazole nucleus, the N–H group at that position allowed for an incremental SI value (*cf.* **3a**–**e** *vs.* **7a**–**e**). Accordingly, the imidazole nucleus bearing an H was considered as an ideal core for conjugation with coumarins.

**Figure 2:**
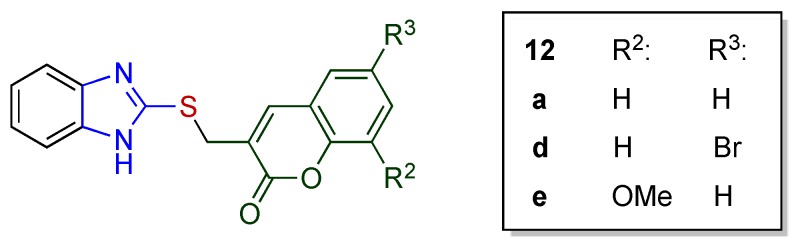
Structure of benzimidazole–coumarin conjugates.

The conjugated compounds with the imidazole nucleus had a higher selectivity than those (*i.e.*, **12a**,**d**,**e**) [11] with a benzimidazole nucleus as shown below (Figure 2). It is due to lower cytotoxicity associated with the conjugates **3a**,**d**,**e** (*cf.* **3a** *vs.* **12a**, **3d** *vs.* **12d** and **3e** *vs.* **12e**). Moreover, the conjugated compounds bearing a simple imidazole nucleus showed greater anti-HCV activity than the corresponding conjugated compounds bearing an inosine or a guanosine nucleus by one order (*cf.* **3** *vs.* **9**, and **3** *vs.* **11**).

## 4. Experimental Section

### 4.1. General Procedures

All reactions were carried out in oven-dried glassware (120 °C) under an atmosphere of nitrogen unless indicated otherwise. Dichloromethane, acetone, and methanol were purchased from Mallinckrodt Chemical Co. (Dublin, Ireland). Acetonitrile was purchased from Fischer Scientific Co. (Hampton, NH, USA). Ethyl acetate (EtOAc) and hexanes from Mallinckrodt Chemical Co. were dried and distilled from CaH_2_. Trimethylsilyl trifluoro-methanesulfonate (Me_3_SiOTf) was purchased from Fluka Chemika (St. Louis, MO, USA). *N*,*O*-Bistrimethylsilylacetamide (BTSA) was purchased from Merck & Co. (Kenilworth, NJ, USA). Aqueous ammonium hydroxide was purchased from J. T. Baker Chemical Co. (Center Valley, PA, USA). *p*-Toluenesulfonic acid monohydrate (PTSA), 1*H*-imidazole-2-thiol (**1a**), and 1-methyl-imidazole-2-thiol (**1b**) were purchased from Sigma-Aldrich Chemical Co. (St. Louis, MO, USA). 1,2,3,5-Tetra-*O*-acetyl-*β*-d-ribofuranose was purchased from Alfa Aesar Chemical Co. (Ward Hill, MA, USA). 3-(Chloromethyl)coumarins [47] **2a**–**e**, 8-mercaptoinosine **8** [40], and 8-mercaptoguanosine **10** [41], were prepared according to the reported methods.

Melting points were obtained with a MP-2D melting point apparatus (Fargo, New Taipei City, Taiwan). Analytical thin layer chromatography (TLC) was performed on pre-coated plates (silica gel 60 F-254) purchased from Merck & Co. (Kenilworth, NJ, USA). Purification by gravity column chromatography was carried out by use of Silicycle (Quebec, QC, Canada) ultra pure silica gel (particle size 40–63 μm, 230–400 mesh). High performance liquid chromatography (HPLC) was performed on two Waters 515 HPLC pumps equipped with a Waters 2489 UV/visible detector (Milford, MA, USA) and a Thermo (Waltham, MA, USA) 5 μm Hypersil ODS (250 mm × 4.6 mm i.d.). Purity of all compounds was >98.0%, as checked by HPLC.

Infrared (IR) spectra were measured on a model Spectrum One B spectrophotometer (Perkin-Elmer, Waltham, MA, USA). Absorption intensities are recorded with the following abbreviations: s, strong; m, medium; w, weak. High-resolution mass spectra were obtained by means of a JMS-700 mass spectrometer (JEOL, Tokyo, Japan). Proton NMR spectra were obtained on a Mercury-400 (400 MHz) spectrometer (Varian, Palo Alto, CA, USA) or an AC-400 (400 MHz) spectrometer (Bruker, Billerica, MA, USA) using chloroform-*d*, or dimethylsulfoxide-*d*_6_ as solvents. Proton NMR chemical shifts are referenced to the CHCl_3_ singlet (δ 7.24 ppm), and the center of DMSO-*d*_6_ quintet (δ 2.49 ppm). Carbon-13 NMR spectra were recorded on a Varian Mercury-400 (100 MHz) spectrometer or Bruker AC-400 (100 MHz) spectrometer using chloroform-*d*, dimethylsulfoxide-*d*_6_, or pyridine-*d*_5_ as solvents. Carbon-13 chemical shifts are referenced to the center of the CDCl_3_ triplet (δ 77.0 ppm), the DMSO-*d*_6_ septet (δ 39.5 ppm), and the pyridine-*d*_5_ triplet (δ 150.4 ppm). Multiplicities are recorded with the following abbreviations: s, singlet; d, doublet; t, triplet; q, quartet; m, multiplet; *J*, coupling constant (hertz).

### 4.2. Standard Procedure for the Synthesis of Imidazole–Coumarin Conjugates ***3***, ***7***, ***9***, and ***11***

To a solution containing a thione (**1**, **6**, **8**, or **10**, 1.0 equiv) in water (2.5 mL) and acetonitrile (1.5 mL) was added saturated aqueous ammonium hydroxide. After the solution was stirred at room temperature for 30 min, a 3-(chloromethyl)coumarin (**2**, 1.5 equiv) was added and stirring was continued at room temperature for 15 min to 2.0 h. Acetonitrile therein was removed under reduced pressure and water was further removed under reduced pressure over P_2_O_5_ with a Kügelrohr GKR-51 apparatus (BUCHI, Flawil, Switzerland). The residue was purified by use of column chromatography packed with silica gel to give the desired products with purity of >98.0%, as determined by HPLC (see Supplementary Materials).

#### 4.2.1. 2-[(Coumarin-3’-yl)methylthio]imidazole (**3a**)

The standard procedure was followed by use of 1*H*-imidazole-2-thiol (**1a**, 28.6 mg, 0.286 mmol, 1.0 equiv), aqueous ammonium hydroxide (0.25 mL), and 3-(chloromethyl)coumarin (**2a**, 66.7 mg, 0.343 mmol, 1.2 equiv). After the solution was stirred at room temperature for 2.0 h and then worked up, the residue was purified by use of column chromatography (5.0% methanol in dichloromethane as the eluent) to give **3a** (63.4 mg, 0.246 mmol) in 86% yield as a white solid: mp (recrystallized from EtOH) 252.0–252.8 °C; ^1^H-NMR (CDCl_3_, 400 MHz) δ 7.58 (s, 1H, O=C–C=CH), 7.39 (s, 2H, 2 × NCH), 7.35–7.29 (m, 2H, 2 × ArH), 7.14–7.10 (m, 2H, 2 × ArH), 4.33 (s, 2H, SCH_2_); ^13^C-NMR (pyridine-*d*_5_, 100 MHz) δ 160.77 (C=O), 153.77, 140.38, 143.12, 139.37, 131.37, 128.18, 126.19, 124.58, 119.72, 116.42, 34.86 (SCH_2_); IR (KBr) 3307 (br, NH), 1713 (s, C=O), 1616 (m), 1587 (m), 1122 (m) cm^−1^; MS (FAB^+^) *m*/*z* 259 (MH**^+^**, 27), 154 (100), 137 (57), 107 (24), 89 (24). HRMS *m*/*z* calcd for C_13_H_10_N_2_O_2_S: 258.0463, found: 258.0462.

#### 4.2.2. 2-[(6′-Fluorocoumarin-3′-yl)methylthio]imidazole (**3b**)

The standard procedure was followed by use of 1*H*-imidazole-2-thiol (**1a**, 29.1 mg, 0.291 mmol, 1.0 equiv), aqueous ammonium hydroxide (0.25 mL), and 3-(chloromethyl)-6-fluorocoumarin (**2b**, 74.2 mg, 0.349 mmol, 1.2 equiv). After the solution was stirred at room temperature for 2.0 h and then worked up, the residue was purified by use of column chromatography (5.0% methanol in dichloromethane as the eluent) to give **3b** (59.9 mg, 0.218 mmol) in 75% yield as a white solid: mp (recrystallized from EtOH) 258.6–259.4 °C; ^1^H-NMR (DMSO-*d*_6_, 400 MHz) δ 7.58 (s, 1H, O=C–C=CH), 7.51–7.43 (m, 3H, 3 × ArH), 7.13 (s, 1H, 1 × NCH), 6.95 (s, 1H, 1 × NCH), 3.99 (s, 2H, SCH_2_); ^13^C-NMR (pyridine-*d*_5_, 100 MHz) δ 160.45 (C=O), 160.01, 157.61, 139.36, 127.33, 125.10, 120.56, 118.62, 118.37, 118.14, 113.54, 113.30, 34.71 (SCH_2_); IR (KBr) 3306 (br, NH), 1713 (s, C=O), 1619 (m), 1504 (m), 1188 (m) cm^−1^; MS (FAB^+^) *m*/*z* 277 (MH**^+^**, 19), 157 (31), 137 (22), 107 (9), 79 (100), 78 (23). HRMS *m*/*z* calcd for C_13_H_9_N_2_O_2_FS: 276.0369, found: 276.0368.

#### 4.2.3. 2-[(6′-Chlorocoumarin-3′-yl)methylthio]imidazole (**3c**)

The standard procedure was followed by use of 1*H*-imidazole-2-thiol (**1a**, 25.6 mg, 0.256 mmol, 1.0 equiv), aqueous ammonium hydroxide (0.25 mL), and 3-(chloromethyl)-6-chlorocoumarin (**2c**, 70.3 mg, 0.307 mmol, 1.2 equiv). After the solution was stirred at room temperature for 2.0 h and then worked up, the residue was purified by use of column chromatography (5.0% methanol in dichloromethane as the eluent) to give **3c** (48.6 mg, 0.166 mmol) in 65% yield as a white solid: mp (recrystallized from EtOH) 266.2–270.0 °C; ^1^H-NMR (DMSO-*d*_6_, 400 MHz) δ 7.73 (s, 1H, ArH), 7.60 (d, 1H, *J* = 8.8 Hz, ArH), 7.57 (s, 1H, O=C—C=CH), 7.43 (d, 1H, *J* = 8.8 Hz, ArH), 7.13 (s, 1H, NCH), 6.96 (s, 1H, NCH), 3.98 (s, 2H, SCH_2_); ^13^C-NMR (pridine-*d*_5_, 100 MHz) δ 160.26 (C=O), 152.08, 139.16, 139.08, 131.06, 129.28, 127.42, 125.11, 122.97, 120.85, 118.05, 34.64 (SCH_2_); IR (KBr) 3309 (br, NH), 1714 (s, C=O), 1615 (m), 1504 (m), 1189 (m) cm^−1^; MS (FAB^+^) *m*/*z* 293 (MH**^+^**, 14), 154 (100), 137 (57), 107 (22), 89 (23), 77 (20). HRMS *m*/*z* calcd for C_13_H_9_N_2_O_2_ClS: 292.0073, found: 292.0074.

#### 4.2.4. 2-[(6′-Bromocoumarin-3′-yl)methylthio]imidazole (**3d**)

The standard procedure was followed by use of 1*H*-imidazole-2-thiol (**1a**, 28.8 mg, 0.288 mmol, 1.0 equiv), aqueous ammonium hydroxide (0.25 mL), and 3-(chloromethyl)-6-bromocoumarin (**2d**, 94.5 mg, 0.345 mmol, 1.2 equiv). After the solution was stirred at room temperature for 2.0 h and then worked up, the residue was purified by use of column chromatography (5.0% methanol in dichloromethane as the eluent) to give **3d** (71.8 mg, 0.213 mmol) in 74% yield as a white solid: mp (recrystallized from EtOH) 276.8–277.4 °C; ^1^H-NMR (CDCl_3_, 400 MHz) δ7.84 (d, *J* = 2.4 Hz, 1H, ArH), 7.71 (dd, *J* = 8.8, 2.4 Hz, 1H, ArH), 7.56 (s, 1H, O=C–C=CH), 7.37 (d, *J* = 8.8 Hz, 1H, ArH), 7.04 (s, 2H, 2 × NCH), 3.98 (s, 2H, SCH_2_); ^13^C-NMR (pyridine-*d*5/DMSO-*d*_6_, 100 MHz) δ 160.23 (C=O), 152.09, 139.40, 137.95, 134.26, 130.28, 126.27, 126.07, 120.93, 118.52, 116.71, 34.38 (SCH_2_); IR (KBr) 3307 (br, NH), 1714 (s, C=O), 1620 (m), 1504 (m), 1189 (m), 1123 (m) cm^−1^; MS (FAB^+^) *m*/*z* 337 (MH**^+^**, 18), 154 (100), 137 (55), 107 (24), 89 (29), 77 (21). HRMS *m*/*z* calcd for C_13_H_9_N_2_O_2_BrS: 335.9568, found: 335.9649.

#### 4.2.5. 2-[(8′-Methoxycoumarin-3′-yl)methylthio]imidazole (**3e**)

The standard procedure was followed by use of 1*H*-imidazole-2-thiol (**1a**, 28.7 mg, 0.287 mmol, 1.0 equiv), aqueous ammonium hydroxide (0.25 mL), and 3-(chloromethyl)-8-methoxycoumarin (**2e**, 76.6 mg, 0.344 mmol, 1.2 equiv). After the solution was stirred at room temperature for 2.0 h and then worked up, the residue was purified by use of column chromatography (5.0% methanol in dichloromethane as the eluent) to give **3e** (66.3 mg, 0.229 mmol) in 80% yield as a white solid: mp (recrystallized from EtOH) 269.8–270.6 °C; ^1^H-NMR (DMSO-*d*_6_, 400 MHz) δ 7.55 (s, 1H, O=C–C=CH), 7.31–7.21 (m, 2H, ArH), 7.10–7.09 (m, 1H, ArH), 7.08 (s, 1H, NCH), 6.97 (s, 1H, NCH), 3.98 (s, 2H, SCH_2_), 3.89 (s 3H, OCH_3_); ^13^C-NMR (pyridine-*d*_5_/DMSO-*d*_6_, 100 MHz) δ 160.45 (C=O), 146.80, 142.83, 140.69, 138.74, 125.60, 124.54, 122.96, 119.91, 119.71, 119.36, 113.62, 55.93 (OCH_3_), 34.52 (SCH_2_); IR (KBr) 3307 (br, NH), 1713 (s, C=O), 1615 (m), 1504 (m), 1189 (m) cm^−1^; MS (FAB^+^) *m*/*z* 289 (MH**^+^**, 15), 154 (100), 137 (52), 107 (28), 89 (23), 77 (24). HRMS *m*/*z* calcd for C_14_H_12_N_2_O_3_S: 288.0569, found: 288.0570.

#### 4.2.6. 1-Methyl-2-[(coumarin-3′-yl)methylthio]imidazole (**3f**)

The standard procedure was followed by use of 1-methyl-imidazole-2-thiol (**1b**, 33.2 mg, 0.291 mmol, 1.0 equiv), aqueous ammonium hydroxide (0.25 mL), and 3-(chloromethyl)coumarin (**2a**, 85.1 mg, 0.437 mmol, 1.5 equiv). After the solution was stirred at room temperature for 2.0 h and then worked up, the residue was purified by use of column chromatography (40% dichloromethane in EtOAc as the eluent) to give **3f** (54.9 mg, 0.201 mmol) in 69% yield as a white solid: mp (recrystallized from EtOH) 244.2–244.8 °C; ^1^H-NMR (CDCl_3_, 400 MHz) δ 7.53 (s, 1H, O=C–C=CH), 7.45 (t, *J* = 7.2 Hz, 1H, ArH), 7.34 (s, *J* = 7.8 Hz, 1H, ArH), 7.28–7.19 (m, 2H, 2 × ArH), 7.05 (s, 1H, NCH), 6.85 (s, 1H, NCH), 4.12 (s, 2H, SCH_2_), 3.47 (s, 3H, NCH_3_); ^13^C-NMR (CDCl_3_, 100 MHz) δ 160.82 (C=O), 153.45, 140.55, 131.3129.47, 127.77, 125.01, 124.45, 122.57, 119.15, 116.67, 116.47, 33.92 (SCH_2_), 33.20 (CH_3_); IR (KBr) 1713 (s, C=O), 1616 (m), 1519 (m), 1170 (m) cm^−1^; MS (FAB^+^) *m*/*z* 272 (M**^+^**, 30), 154 (100), 136 (77), 89 (33), 77 (20), 55 (60); HRMS *m*/*z* calcd for C_14_H_12_N_2_O_2_S: 272.0619, found: 272.0620.

#### 4.2.7. 1-Methyl-2-[(6′-chlorocoumarin-3′-yl)methylthio]imidazole (**3g**)

The standard procedure was followed by use of 1-methyl-imidazole-2-thiol (**1b**, 36.2 mg, 0.317 mmol, 1.0 equiv), aqueous ammonium hydroxide (0.25 mL), and 3-(chloromethyl)-6-chlorocoumarin (**2c**, 109 mg, 0.476 mmol, 1.5 equiv). After the solution was stirred at room temperature for 2.0 h and then worked up, the residue was purified by use of column chromatography (40% dichloromethane in EtOAc as the eluent) to give **3g** (70.5 mg, 0.231 mmol) in 73% yield as a white solid: mp (recrystallized from EtOH) 256.2–257.2 °C; ^1^H-NMR (CDCl_3_, 400 MHz) δ 7.51 (s, 1H, O=C–C=CH), 7.40 (d, *J* = 8.8 Hz, 1H, ArH), 7.34 (s, 1H, ArH), 7.22 (d, *J* = 8.8 Hz, 1H, ArH), 7.06 (s, 1H, NCH), 6.87 (s, 1H, NCH), 4.12 (s, 2H, SCH_2_), 3.49 (s, 3H, NCH_3_); ^13^C-NMR (CDCl_3_, 100 MHz) δ 160.27 (C=O), 151.80, 140.40, 139.27, 131.23, 129.73, 129.49, 126.98, 126.38, 122.66, 120.22, 117.92, 33.56 (SCH_2_), 33.20 (CH_3_); IR (KBr) 1705 (s, C=O), 1615 (m), 1518 (m), 1233 (m) cm^−1^; MS (FAB^+^) *m*/*z* 307 (MH**^+^**, 100), 154 (66), 136 (59), 77 (30), 55 (35), 77 (20); HRMS *m*/*z* calcd for C_14_H_11_N_2_O_2_ClS: 306.0230, found: 306.0233.

#### 4.2.8. 1-(2′,3′,5′-Tri-*O*-acetyl-β-d-ribofuranos-1′-yl)imidazole-2-thiol (**5**)

To a solution of 1*H*-imidazole-2-thiol (**1a**, 375 mg, 3.74 mmol, 1.0 equiv) in dry acetonitrile (30 mL) was added BTSA (1.20 mL, 4.91 mmol, 1.3 equiv) under nitrogen atmosphere according to the Vorbrüggen procedure [43]. After the solution was stirred at 60 °C for 30 min, 1,2,3,5-tetra-*O*-acetyl-β-d-ribofuranose (**4**, 1.25 g, 3.93 mmol, 1.1 equiv) and Me_3_SiOTf (0.711 mL, 3.93 mmol, 1.1 equiv) were added in sequence. The reaction mixture was heated to 80 °C and stirred for 18 h. Excess solvent was removed under reduced pressure, and the residue was treated with 20% aqueous NaHCO_3_ solution (30 mL). The aqueous layer was extracted with ethyl acetate (3 × 25 mL). The combined organic layers were washed with brine (30 mL), dried over MgSO_4_ (s), filtered, and concentrated under reduced pressure to afford a residue, which was purified by use of column chromatography (20% hexanes in EtOAc as the eluent) to give **5** (698.4 mg, 1.945 mmol) in 52% yield. ^1^H-NMR (CDCl_3_, 400 MHz) δ 6.90 (s, 1H, NCH), 6.72 (s, 1H, NCH), 6.47 (s, 1H, H-1″), 5.44 (t, *J* = 5.2 Hz, 1H, H-2″), 5.31 (t, *J* = 5.2 Hz, 1H, H-3″), 4.38–4.36 (m, 1H, H-4″), 4.33–4.28 (m, 2H, 2 × H-5″), 2.13 (s, 3H, CO_2_CH_3_), 2.11 (s, 3H, CO_2_CH_3_), 2.09 (s, 3H, CO_2_CH_3_). These data are consistent with those reported [48].

#### 4.2.9. 1-β-d-Ribofuranosyl-imidazole-2-thiol (**6**)

To a solution containing **5** (82.6 mg, 0.230 mmol, 1.0 equiv) in methanol (10 mL) was added potassium carbonate (31.9 mg, 0.231 mmol, 1.0 equiv) at room temperature. After the solution was stirred at 0 °C for 12 h, it was concentrated under reduced pressure. To the residue was added saturated aqueous NH_4_Cl (20 mL), which was extracted with CHCl_3_ (3 × 20 mL). The combined organic layers were washed with brine (20 mL), dried over MgSO_4_ (s), filtered, and concentrated under reduced pressure. The residue was then purified by use of column chromatography (10% MeOH in EtOAc as the eluent) to give **6** (49.1 mg, 0.212 mmol) in 92% yield. ^1^H-NMR (DMSO-*d*_6_, 400 MHz) δ 7.29 (s, 1H, NCH), 6.91 (s, 1H, NCH), 6.02 (s, 1H, H-1″), 5.27 (d, *J* = 6.6 Hz, 1H, OH), 5.03 (d, *J* = 4.0 Hz, 1H, OH), 4.98 (t, *J* = 5.2 Hz, 1H, OH), 4.07–4.04 (m, 1H, H-2″), 4.03–3.99 (m, 1H, H-3″), 3.83–3.81 (m, 1H, H-4″), 3.63–3.53 (m, 2H, 2 × H-5″). These data are consistent with those reported [48].

#### 4.2.10. 1-(β-d-Ribofuranos-1″-yl)-2-[(coumarin-3′-yl)methylthio]imidazole (**7a**)

The standard procedure was followed by use of 1-β-d-ribofuranosyl-imidazole-2-thiol (**6**, 43.1 mg, 0.186 mmol, 1.0 equiv), aqueous ammonium hydroxide (0.25 mL), and 3-(chloromethyl)coumarin (**2a**, 43.4 mg, 0.223 mmol, 1.2 equiv). After the solution was stirred at room temperature for 2.0 h and then worked up, the residue was purified by use of column chromatography (10% MeOH in dichloromethane as the eluent) to give **7a** (53.7 mg, 0.165 mmol) in 74% yield as a white solid: mp (recrystallized from EtOH) 164.6–165.4 °C; ^1^H-NMR (DMSO-*d*_6_, 400 MHz) δ 7.66 (s, 1H, O=C–C=CH), 7.58–7.54 (m, 2H, 2 × ArH), 7.49 (s, 1H, NCH), 7.38 (d, *J* = 8.4 Hz, 1H, ArH), 7.30 (t, *J* = 7.6 Hz, 1H, ArH), 6.99 (s, 1H, NCH), 5.67 (d, *J* = 7.2 Hz, 1H, H-1″), 5.37 (d, *J* = 6.4 Hz, 1H, OH), 5.17 (d, *J* = 4.4 Hz, 1H, OH), 4.93 (t, *J* = 5.2 Hz, 1H, OH), 4.16–4.11 (m, 1H, H-2″), 4.04 (s, 2H, SCH_2_), 3.99–3.98 (m, 1H, H-3″), 3.78–3.76 (m, 1H, H-4″), 3.43–3.39 (m, 2H, 2 × H-5″); ^13^C-NMR (DMSO-*d*_6_, 100 MHz) δ 160.03 (C=O), 151.94, 140.67, 139.65, 131.57, 129.62, 128.20, 124.59, 124.44, 119.54, 118.97, 116.00, 88.13, 85.58, 75.10, 70.55, 61.36, 34.27 (SCH_2_); IR (KBr) 3387 (br, OH), 1698 (s, C=O), 1609 (m), 1457 (m), 1111 (m) cm^−1^; MS (FAB^+^) *m*/*z* 391 (MH**^+^**, 66), 154 (100), 137 (54), 77 (33); HRMS *m*/*z* calcd for C_18_H_18_N_2_O_6_S: 390.0886, found: 390.0891.

#### 4.2.11. 1-(β-d-Ribofuranos-1″-yl)-2-[(6′-fluorocoumarin-3′-yl)methylthio]imidazole (**7b**)

The standard procedure was followed by use of 1-β-d-ribofuranosyl-imidazole-2-thiol (**6**, 44.9 mg, 0.193 mmol, 1.0 equiv), aqueous ammonium hydroxide (0.25 mL), and 3-(chloromethyl)-6-fluorocoumarin (**2b**, 49.3 mg, 0.232 mmol, 1.2 equiv). After the solution was stirred at room temperature for 2.0 h and then worked up, the residue was purified by use of column chromatography (10% MeOH in dichloromethane as the eluent) to give **7b** (62.9 mg, 0.154 mmol) in 80% yield as a white solid: mp (recrystallized from EtOH) 171.2–172.4 °C; ^1^H-NMR (DMSO-*d*_6_, 400 MHz) δ 7.62 (s, 1H, O=C–C=CH), 7.50 (s, 1H, ArH), 7.47–7.42 (m, 3H, 2 × ArH + NCH), 7.00 (s, 1H, NCH), 5.65 (d, *J* = 6.4 Hz, 1H, H-1″), 5.36 (d, *J* = 6.8 Hz, 1H, OH), 5.18 (d, *J* = 7.6 Hz, 1H, OH), 4.94 (t, *J* = 5.2 Hz, 1H, OH), 4.16–4.11 (m, 1H, H-2″), 4.03 (s, 2H, SCH_2_), 4.00–3.97 (m, 1H, H-3″), 3.77–3.74 (m, 1H, H-4″), 3.42–3.38 (m, 2H, 2 × H-5″); ^13^C-NMR (CDCl_3_, 100 MHz) δ 159.73 (C=O), 149.28, 139.60, 139.51, 129.64, 125.67, 119.97, 119.52, 118.78, 118.54, 117.96, 113.49, 88.08, 85.54, 75.01, 70.49, 61.32, 34.22 (SCH_2_); IR (KBr) 3388 (br, OH), 1694 (s, C=O), 1580 (m), 1489 (m), 1265 (m), 1078 (m) cm^−1^; MS (FAB^+^) *m*/*z* 409 (MH**^+^**, 54), 154 (100), 137 (57), 77 (31), 55 (66); HRMS *m*/*z* calcd for C_18_H_17_N_2_O_6_FS: 408.0791, found: 408.0794.

#### 4.2.12. 1-(β-d-Ribofuranos-1″-yl)-2-[(6′-chlorocoumarin-3′-yl)methylthio]imidazole (**7c**)

The standard procedure was followed by use of 1-β-d-ribofuranosyl-imidazole-2-thiol (**6**, 44.6 mg, 0.192 mmol, 1.0 equiv), aqueous ammonium hydroxide (0.25 mL), and 3-(chloromethyl)-6-chlorocoumarin (**2c**, 52.8 mg, 0.231 mmol, 1.2 equiv). After the solution was stirred at room temperature for 2.0 h and then worked up, the residue was purified by use of column chromatography (10% MeOH in dichloromethane as the eluent) to give **7c** (61.2 mg, 0.144 mmol) in 75% yield as a white solid: mp (recrystallized from EtOH) 178.8–179.6 °C; ^1^H-NMR (DMSO-*d*_6_, 400 MHz) δ 7.69 (s, 1H, O=C–C=CH), 7.60 (s, 1H, ArH), 7.59–7.57 (m, 2H, 2 × ArH), 7.49 (s, 1H, NCH), 7.41 (d, *J* = 8.8 Hz, 1H, ArH), 7.00 (s, 1H, NCH), 5.65 (d, *J* = 6.4 Hz, 1H, H-1″), 5.34 (d, *J* = 6.4 Hz, 1H, OH), 5.16 (d, *J* = 4.8 Hz, 1H, OH), 4.92 (t, *J* = 5.2 Hz, 1H, OH), 4.15–4.11 (m, 1H, H-2″), 4.02 (s, 2H, SCH_2_), 4.00–3.97 (m, 1H, H-3″), 3.77–3.74 (m, 1H, H-4″), 3.41–3.38 (m, 2H, 2 × H-5″); ^13^C-NMR (CDCl_3_, 100 MHz) δ 159.53 (C=O), 151.54, 139.44, 139.31, 131.05, 129.67, 128.27, 127.17, 125.75, 120.38, 119.54, 117.98, 88.07, 85.55, 75.01, 70.50, 61.31, 34.31 (SCH_2_); IR (KBr) 3399 (br, OH), 1695 (s, C=O), 1615 (m), 1580 (m), 1441 (m), 1265 (m), 1173 (m) cm^−1^; MS (FAB^+^) *m*/*z* 425 (MH**^+^**, 55), 154 (100), 137 (53), 77 (35), 55 (61); HRMS *m*/*z* calcd for C_18_H_17_N_2_O_6_ClS: 424.0496, found: 424.0580.

#### 4.2.13. 1-(β-d-Ribofuranos-1″-yl)-2-[(6′-bromocoumarin-3′-yl)methylthio]imidazole (**7d**)

The standard procedure was followed by use of 1-β-d-ribofuranosyl-imidazole-2-thiol (**6**, 47.2 mg, 0.203 mmol, 1.0 equiv), aqueous ammonium hydroxide (0.25 mL), and 3-(chloromethyl)-6-bromocoumarin (**2d**, 66.7 mg, 0.244 mmol, 1.2 equiv). After the solution was stirred at room temperature for 2.0 h and then worked up, the residue was purified by use of column chromatography (10% MeOH in dichloromethane as the eluent) to give **7d** (79.8 mg, 0.171 mmol) in 84% yield as a white solid: mp (recrystallized from EtOH) 188.8–189.4 °C; ^1^H-NMR (DMSO-*d*_6_, 400 MHz) δ 7.82 (s, 1H, O=C–C=CH), 7.72–7.69 (m, 1H, ArH), 7.60 (s, 1H, ArH), 7.50 (s, 1H, NCH), 7.36 (d, *J* = 8.8 Hz, 1H, ArH), 7.01 (s, 1H, NCH), 5.65 (d, *J* = 7.2 Hz, 1H, H-1″), 5.36 (d, *J* = 6.8 Hz, 1H, OH), 5.17 (d, *J* = 4.4 Hz, 1H, OH), 4.93 (t, *J* = 5.2 Hz, 1H, OH), 4.16–4.11 (m, 1H, H-2″), 4.02 (s, 2H, SCH_2_), 4.00–3.97 (m, 1H, H-3″), 3.76–3.74 (m, 1H, H-4″), 3.41–3.38 (m, 2H, 2 × H-5″); ^13^C-NMR (CDCl_3_, 100 MHz) δ 159.46 (C=O), 151.95, 139.39, 139.23, 133.82, 130.13, 129.63, 125.68, 120.86, 119.56, 118.25, 116.09, 88.07, 85.56, 75.02, 70.50, 61.30, 34.33 (SCH_2_); IR (KBr) 3400 (br, OH), 1693 (s, C=O), 1599 (m), 1479 (m), 1399 (m), 1263 (m), 1172 (m) cm^−1^; MS (FAB^+^) *m*/*z* 469 (MH^+^, 45), 154 (100), 137 (55), 77 (32), 55 (60); HRMS *m*/*z* calcd for C_18_H_17_N_2_O_6_BrS: 467.9991, found: 468.0072.

#### 4.2.14. 1-(β-d-Ribofuranos-1″-yl)-2-[(8′-methoxycoumarin-3′-yl)methylthio]imidazole (**7e**)

The standard procedure was followed by use of 1-β-d-ribofuranosyl-imidazole-2-thiol (**6**, 46.8 mg, 0.201 mmol, 1.0 equiv), aqueous ammonium hydroxide (0.25 mL), and 3-(chloromethyl)-8-methoxycoumarin (**2e**, 53.8 mg, 0.241 mmol, 1.2 equiv). After the solution was stirred at room temperature for 2.0 h and then worked up, the residue was purified by use of column chromatography (10% MeOH in dichloromethane as the eluent) to give **7e** (61.2 mg, 0.145 mmol) in 72% yield as a white solid: mp (recrystallized from EtOH) 169.6–170.4 °C; ^1^H-NMR (DMSO-*d*_6_, 400 MHz) δ 7.63 (s, 1H, O=C–C=CH), 7.49 (s, 1H, NCH), 7.25–7.20 (m, 2H, 2 × ArH), 7.10–7.08 (m, 1H, ArH), 6.99 (s, 1H, NCH), 5.66 (d, *J* = 6.0 Hz, 1H, H-1″), 5.38 (d, *J* = 6.8 Hz, 1H, OH), 5.18 (d, *J* = 4.4 Hz, 1H, OH), 4.94 (t, *J* = 5.2 Hz, 1H, OH), 4.16–4.11 (m, 1H, H-2″), 4.07 (s, 2H, SCH_2_), 4.03–3.97 (m, 1H, H-3″), 3.88 (s, 3H, OCH_3_), 3.79–3.76 (m, 1H, H-4″), 3.43–3.40 (m, 2H, 2 × H-5″); ^13^C-NMR (DMSO-*d*_6_, 100 MHz) δ 159.71 (C=O), 146.33, 142.24, 140.85, 139.58, 129.57, 124.60, 124.50, 119.49, 119.36, 113.86, 88.10, 85.54, 75.05, 70.49, 61.33, 56.08 (OCH_3_), 34.14 (SCH_2_); IR (KBr) 3403 (br, OH), 1695 (s, C=O), 1579 (m), 1402 (m), 1265 (m), 1102 (m), 1078 (m) cm^−1^; MS (FAB^+^) *m*/*z* 421 (MH**^+^**, 15), 154 (100), 137 (30), 77 (66), 55 (67); HRMS *m*/*z* calcd for C_19_H_20_N_2_O_7_S: 420.0991, found: 420.0987.

#### 4.2.15. 8-[(Coumarin-3′-yl)methylthio]inosine (**9a**)

The standard procedure was followed by use of **8** (48.7 mg, 0.162 mmol, 1.0 equiv), water (2.5 mL), acetonitrile (1.5 mL), aqueous ammonium hydroxide (0.15 mL), and 3-(chloromethyl)coumarin (**2a**, 37.8 mg, 0.194 mmol, 1.2 equiv). It was stirred at room temperature for 30 min and then worked up. The residue was purified by use of column chromatography (10% methanol in CH_2_Cl_2_ as the eluent) to give **9a** (52.4 mg, 0.114 mmol) in 70% yield as a white solid: mp (recrystallized from dichloromethane/methanol) 160.9–162.5 °C; ^1^H-NMR (DMSO-*d*_6_, 400 MHz) δ 8.06 (s, 1H, H-2), 8.02 (s, 1H, CH=C–COO), 7.65–7.57 (m, 2H, 2 × ArH), 7.41 (d, *J* = 8.4 Hz, 1H, ArH), 7.33 (dd, *J* = 7.6, 7.2 Hz, 1H, ArH), 5.76 (d, *J* = 6.4 Hz, 1H, H-1″), 5.41 (d, *J* = 5.6 Hz, 1H, OH), 5.19 (d, *J* = 3.6 Hz, 1H, OH), 5.01–4.99 (br, 1H, OH), 4.89–4.85 (m, 1H, H-2″), 4.39 (d, *J* = 14.2 Hz, 1H, SCH), 4.35 (d, *J* = 14.2 Hz, 1H, SCH), 4.12–4.09 (m, 1H, H-3″), 3.88–3.85 (m, 1H, H-4″), 3.63–3.57 (m, 1H, H-5″), 3.50–3.44 (m, 1H, H-5″); ^13^C-NMR (DMSO-*d*_6_, 100 MHz) δ 160.07 (C=O), 155.37 (C=O), 152.89, 149.57, 146.63, 145.28, 141.20, 131.76, 128.37, 124.90, 124.60, 123.84, 118.74, 116.10, 88.74, 86.06, 71.16, 70.50, 61.81, 32.04 (SCH_2_); IR (ATR) 3359 (br, OH + NH), 1707 (s, C=O), 1678 (s, C=O), 1583 (m), 1189 (m), 1051 (s) cm^−1^; MS (FAB^+^) *m*/*z* 459 (MH^+^, 2), 327 (4), 221 (13), 147 (13), 55 (100); HRMS (FAB) calcd for (C_20_H_18_N_4_O_7_S + H)^+^: 459.0974, found: 459.0984.

#### 4.2.16. 8-[(6′-Fluorocoumarin-3′-yl)methylthio]inosine (**9b**)

The standard procedure was followed by use of **8** (56.7 mg, 0.189 mmol, 1.0 equiv), water (2.5 mL), acetonitrile (1.5 mL), aqueous ammonium hydroxide (0.15 mL), and 3-(chloromethyl)-6-fluorocoumarin (**2b**, 48.2 mg, 0.227 mmol, 1.2 equiv). It was stirred at room temperature for 30 min and then worked up. The residue was purified by use of column chromatography (10% methanol in CH_2_Cl_2_ as the eluent) to give **9b** (74.4 mg, 0.156 mmol) in 83% yield as a white solid: mp (recrystallized from dichloromethane/methanol) 168.3–169.1 °C; ^1^H-NMR (DMSO-*d*_6_, 400 MHz) δ 12.50–12.42 (br, 1H, NH), 8.03 (s, 1H, H-2), 8.01 (s, 1H, CH=C–COO), 7.56–7.54 (m, 1H, ArH), 7.48–7.46 (m, 2H, 2 × ArH), 5.76 (d, *J* = 6.4 Hz, 1H, H-1″), 5.43 (d, *J* = 6.0 Hz, 1H, OH), 5.23 (d, *J* = 4.4 Hz, 1H, OH), 5.01–4.99 (br, 1H, OH), 4.89–4.85 (m, 1H, H-2″), 4.39 (d, *J* = 14.2 Hz, 1H, SCH), 4.34 (d, *J* = 14.2 Hz, 1H, SCH), 4.12–4.08 (m, 1H, H-3″), 3.88–3.84 (m, 1H, H-4″), 3.62–3.57 (m, 1H, H-5″), 3.50–3.44 (m, 1H, H-5″); ^13^C-NMR (DMSO-*d*_6_, 100 MHz) δ 159.86 (C=O), 158.08 (d, C=O), 155.40, 149.60, 149.28, 146.51, 145.35, 140.09, 125.18, 124.99, 119.75 (d), 118.95 (d), 118.05 (d), 113.56 (d), 88.79, 86.15, 71.29, 70.45, 61.83, 32.09 (SCH_2_); IR (ATR) 3359 (br, OH + NH), 1706 (s, C=O), 1676 (s, C=O), 1583 (m), 1452 (m), 1188 (m), 1061 (s) cm^−1^; MS (FAB^+^) *m*/*z* 477 (MH^+^, 25), 345 (65), 289 (45), 154 (100), 120 (49); HRMS (FAB) calcd for (C_20_H_17_FN_4_O_7_S + H)^+^: 477.0880, found: 477.0877.

#### 4.2.17. 8-[(6′-Bromocoumarin-3′-yl)methylthio]inosine (**9d**)

The standard procedure was followed by use of **8** (45.8 mg, 0.153 mmol, 1.0 equiv), water (2.5 mL), acetonitrile (1.5 mL), aqueous ammonium hydroxide (0.15 mL), and 6-bromo-3-(chloromethyl)coumarin (**2d**, 50.2 mg, 0.184 mmol, 1.2 equiv). It was stirred at room temperature for 20 min and then worked up. The residue was purified by use of column chromatography (10% methanol in CH_2_Cl_2_ as the eluent) to give **9d** (66.4 mg, 0.126 mmol) in 80% yield as a white solid: mp (recrystallized from dichloromethane/methanol) 169.1–170.5 °C; ^1^H-NMR (DMSO-*d*_6_, 400 MHz) δ 11.95–11.92 (br, 1H, NH), 8.04 (s, 1H, H-2), 8.00 (s, 1H, CH=C–COO), 7.90 (d, *J* = 2.4 Hz, 1H, ArH), 7.73 (dd, *J* = 8.8, 2.4 Hz, 1H, ArH), 7.38 (d, *J* = 8.8 Hz, 1H, ArH), 5.76 (d, *J* = 6.8 Hz, 1H, H-1″), 5.42–5.40 (br, 1H, OH), 5.22–5.20 (br, 1H, OH), 4.96–4.94 (br, 1H, OH), 4.89–4.86 (m, 1H, H-2″), 4.38 (d, *J* = 14.4 Hz, 1H, SCH), 4.34 (d, *J* = 14.4 Hz, 1H, SCH), 4.12–4.09 (m, 1H, H-3″), 3.88–3.85 (m, 1H, H-4″), 3.62–3.59 (m, 1H, H-5″), 3.50–3.45 (m, 1H, H-5″); ^13^C-NMR (DMSO-*d*_6_, 100 MHz) δ 159.61 (C=O), 155.36 (C=O), 151.93, 149.58, 146.45, 145.32, 139.72, 134.08, 130.32, 125.19, 124.98, 120.66, 118.36, 116.21, 88.79, 86.15, 71.29, 70.45, 61.83, 32.09 (SCH_2_); IR (ATR) 3356 (br, OH + NH), 1708 (s, C=O), 1678 (s, C=O), 1585 (m), 1188 (m), 1022 (s) cm^−1^; MS (FAB^+^) *m*/*z* 537 (MH^+^, 2), 427 (13), 307 (19), 185 (26), 154 (100); HRMS (FAB) calcd for (C_20_H_17_BrN_4_O_7_S + H)^+^: 537.0080, found: 537.0077.

#### 4.2.18. 8-[(8′-Methoxycoumarin-3′-yl)methylthio]inosine (**9e**)

The standard procedure was followed by use of **8** (35.8 mg, 0.119 mmol, 1.0 equiv), water (2.5 mL), acetonitrile (1.5 mL), aqueous ammonium hydroxide (0.15 mL), and 3-chloromethyl-8-methoxycoumarin (**2e**, 41.9 mg, 0.201 mmol, 1.2 equiv). It was stirred at room temperature for 30 min and then worked up. The residue was purified by use of column chromatography (10% methanol in CH_2_Cl_2_ as the eluent) to give **9e** (46.7 mg, 95.6 μmol) in 80% yield as a white solid: mp (recrystallized from dichloromethane/methanol) 162.3–164.1 °C; ^1^H-NMR (DMSO-*d*_6_, 400 MHz) δ 12.52–12.39 (br, 1H, NH), 8.04 (s, 1H, H-2), 8.03 (s, 1H, CH=C–COO), 7.28–7.23 (m, 2H, 2 × ArH), 7.18–7.16 (m, 1H, ArH), 5.75 (d, *J* = 6.4 Hz, 1H, H-1″), 5.45–5.41 (br, 1H, OH), 5.23–5.21 (br, 1H, OH), 4.96 (dd, *J* = 6.0, 5.6 Hz, 1H, OH), 4.88–4.85 (m, 1H, H-2″), 4.39 (d, *J* = 14.0 Hz, 1H, SCH), 4.34 (d, *J* = 14.0 Hz, 1H, SCH), 4.13–4.09 (m, 1H, H-3″), 3.89 (s, 3H, OCH_3_), 3.87–3.85 (m, 1H, H-4″), 3.62–3.59 (m, 1H, H-5″), 3.49–3.46 (m, 1H, H-5″); ^13^C-NMR (DMSO-*d*_6_, 100 MHz) δ 159.83 (C=O), 155.39 (C=O), 149.60, 146.69, 146.39, 145.31, 142.24, 141.15, 124.96, 124.67, 124.03, 119.49, 119.32, 114.13, 88.76, 86.15, 71.28, 70.46, 61.83, 56.10 (OCH_3_), 32.01 (SCH_2_); IR (ATR) 3354 (br, OH + NH), 1708 (s, C=O), 1692 (s, C=O), 1583 (m), 1189 (m), 1060 (s) cm^−1^; MS (ESI) *m*/*z* 488 (M^+^); HRMS (ESI) calcd for (C_21_H_20_N_4_O_8_S)^+^: 488.1002, found: 488.1011.

#### 4.2.19. 8-[(6′-Methylcoumarin-3′-yl)methylthio]inosine (**9f**)

The standard procedure was followed by use of **8** (50.2 mg, 0.167 mmol, 1.0 equiv), water (2.5 mL), acetonitrile (1.5 mL), aqueous ammonium hydroxide (0.15 mL), and 3-chloromethyl-6-methylcoumarin (**2f**, 41.9 mg, 0.201 mmol, 1.2 equiv). It was stirred at room temperature for 15 min and then worked up. The residue was purified by use of column chromatography (10% methanol in CH_2_Cl_2_ as the eluent) to give **9f** (64.6 mg, 0.137 mmol) in 82% yield as a white solid: mp (recrystallized from dichloromethane/methanol) 156.8–158.4 °C; ^1^H-NMR (DMSO-*d*_6_, 400 MHz) δ 12.48–12.38 (br, 1H, NH), 8.03 (s, 1H, H-2), 8.01 (s, 1H, CH=C–COO), 7.41–7.38 (m, 2H, 2 × ArH), 7.30 (d, *J* = 8.4 Hz, 1H, ArH), 5.75 (d, *J* = 6.4 Hz, 1H, H-1″), 5.43 (d, *J* = 5.2 Hz, 1H, OH), 5.22 (d, *J* = 2.4 Hz, 1H, OH), 4.95 (dd, *J* = 6.0, 5.6 Hz, 1H, OH), 4.88–4.86 (m, 1H, H-2″), 4.38 (d, *J* = 14.0 Hz, 1H, SCH), 4.34 (d, *J* = 14.0 Hz, 1H, SCH), 4.12–4.09 (m, 1H, H-3″), 3.89–3.85 (m, 1H, H-4″), 3.64–3.58 (m, 1H, H-5″), 3.51–3.48 (m, 1H, H-5″), 2.32 (s, 3H, CH_3_); ^13^C-NMR (DMSO-*d*_6_, 100 MHz) δ 160.26 (C=O), 155.37 (C=O), 151.07, 149.62, 146.77, 145.27, 141.16, 134.00, 132.66, 127.95, 124.95, 123.78, 118.50, 115.86, 88.76, 86.15, 71.29, 70.45, 61.85, 32.00 (SCH_2_), 20.17 (CH_3_); IR (ATR) 3345 (br, OH + NH), 1707 (s, C=O), 1692 (s, C=O), 1583 (m), 1188 (m), 1023 (s) cm^−1^; MS (FAB^+^) *m*/*z* 473 (MH^+^, 1), 341 (10), 185 (16), 109 (30), 55 (100); HRMS (FAB) calcd for (C_21_H_20_N_4_O_7_S)^+^: 472.1053, found: 472.1060.

#### 4.2.20. 8-[(6′-Fluorocoumarin-3′-yl)methylthio]guanosine (**11b**)

The standard procedure was followed by use of **10** (65.2 mg, 0.207 mmol, 1.0 equiv), water (5.0 mL), acetonitrile (3.0 mL), aqueous ammonium hydroxide (0.25 mL), and 3-chloromethyl-6-fluorocoumarin (**2b**, 52.8 mg, 0.248 mmol, 1.2 equiv). It was stirred at room temperature for 15 min and then worked up. The residue was purified by use of column chromatography (15% methanol in CH_2_Cl_2_ as the eluent) to give **11b** (90.2 mg, 0.183 mmol) in 89% yield as a white solid: mp (recrystallized from dichloromethane/methanol) 184.9–186.2 °C; ^1^H-NMR (DMSO-*d*_6_, 400 MHz) δ 10.70–10.66 (br, 1H, NH), 7.90 (s, 1H, CH=C–COO), 7.53–7.42 (m, 3H, 3 × ArH), 6.41 (s, 2H, NH_2_), 5.70 (d, *J* = 6.8 Hz, 1H, H-1″), 5.35 (d, *J* = 6.0 Hz, 1H, OH), 5.07 (d, *J* = 4.8 Hz, 1H, OH), 4.94 (dd, *J* = 6.8, 5.2 Hz, 1H, OH), 4.89–4.85 (m, 1H, H-2″), 4.24 (d, *J* = 14.0 Hz, 1H, SCH), 4.19 (d, *J* = 14.0 Hz, 1H, SCH), 4.10–4.06 (m, 1H, H-3″), 3.80–3.76 (m, 1H, H-4″), 3.62–3.56 (m, 1H, H-5″), 3.49–3.43 (m, 1H, H-5″); ^13^C-NMR (DMSO-*d*_6_, 100 MHz) δ 159.83 (C=O), 158.07 (d, C=O), 155.60, 153.10, 152.47, 149.25, 141.48, 139.84, 125.44, 119.81 (d), 118.83 (d), 118.00 (d), 117.42, 113.47 (d), 88.27, 85.74, 70.53, 61.96, 32.77 (SCH_2_); IR (ATR) 3146 (br, OH + NH), 1723 (m, C=O), 1681 (s, C=O), 1626 (s), 1071(s) cm^−1^; MS (FAB^+^) *m*/*z* 492 (MH^+^, 35), 360 (62), 307 (98), 219 (23), 156 (100), 107 (100); HRMS (FAB) calcd for (C_20_H_18_FN_5_O_7_S + H)^+^: 492.0989, found: 492.0996.

#### 4.2.21. 8-[(8′-Methoxycoumarin-3′-yl)methylthio]guanosine (**11e**)

The standard procedure was followed by use of **10** (63.7 mg, 0.202 mmol, 1.0 equiv), water (5.0 mL), acetonitrile (3.0 mL), aqueous ammonium hydroxide (0.25 mL), and 3-chloromethyl-8-methoxycoumarin (**2e**, 54.0 mg, 0.243 mmol, 1.2 equiv). It was stirred at room temperature for 1.0 h and then worked up. The residue was purified by use of column chromatography (10% methanol in CH_2_Cl_2_ as the eluent) to give **11e** (84.3 mg, 0.167 mmol) in 83% yield as a white solid: mp (recrystallized from dichloromethane/methanol) 192.8–194.3 °C; ^1^H-NMR (DMSO-*d*_6_, 400 MHz) δ 10.68–10.64 (br, 1H, NH), 7.93 (s, 1H, CH=C–COO), 7.28–7.23 (m, 2H, 2 × ArH), 7.16–7.14 (m, 1H, ArH), 6.42 (s, 2H, NH_2_), 5.69 (d, *J* = 6.8 Hz, 1H, H-1″), 5.38 (d, *J* = 6.4 Hz, 1H, OH), 5.09 (d, *J* = 5.2 Hz, 1H, OH), 4.95 (dd, *J* = 6.0, 5.6 Hz, 1H, OH), 4.88–4.84 (m, 1H, H-2″), 4.24 (d, *J* = 13.8 Hz, 1H, SCH), 4.19 (d, *J* = 13.8 Hz, 1H, SCH), 4.09–4.05 (m, 1H, H-3″), 3.89 (s, 3H, OCH_3_), 3.80–3.77 (m, 1H, H-4″), 3.61–3.56 (m, 1H, H-5″), 3.49–3.43 (m, 1H, H-5″); ^13^C-NMR (DMSO-*d*_6_, 100 MHz) δ 159.87 (C=O), 155.68 (C=O), 153.14, 152.55, 146.41, 142.25, 141.73, 141.24, 124.66, 124.34, 119.47, 119.41, 117.38, 114.05, 88.26, 85.78, 70.53, 62.01, 56.16 (OCH_3_), 32.63 (SCH_2_); IR (ATR) 3142 (br, OH + NH), 1714 (m, C=O), 1682 (s, C=O), 1274 (m), 1092 (m) cm^−1^; MS (FAB^+^) *m*/*z* 503 (M^+^, 2), 372 (10), 232 (11), 154 (45), 79 (100); HRMS (FAB) calcd for (C_21_H_21_N_5_O_8_S)^+^: 503.1107, found: 503.1111.

#### 4.2.22. 8-[(6′-Methylcoumarin-3′-yl)methylthio]guanosine (**11f**)

The standard procedure was followed by use of **10** (52.3 mg, 0.166 mmol, 1.0 equiv), water (5.0 mL), acetonitrile (3.0 mL), aqueous ammonium hydroxide (0.25 mL), and 3-chloromethyl-6-methylcoumarin (**2f**, 41.5 mg, 0.199 mmol, 1.2 equiv). It was stirred at room temperature for 30 min and then worked up. The residue was purified by use of column chromatography (15% methanol in CH_2_Cl_2_ as the eluent) to give **11f** (59.6 mg, 0.122 mmol) in 74% yield as a white solid: ^1^H-NMR (DMSO-*d*_6_, 400 MHz) δ 10.74–10.70 (br, 1H, NH), 7.90 (s, 1H, CH=C–COO), 7.40–7.38 (m, 2H, 2 × ArH), 7.30 (d, *J* = 8.8 Hz, 1H, ArH), 6.46 (s, 2H, NH_2_), 5.69 (d, *J* = 6.4 Hz, 1H, H-1″), 5.38 (d, *J* = 6.4 Hz, 1H, OH), 5.09 (d, *J =* 4.8 Hz, 1H, OH), 4.95 (dd, *J* = 6.0, 6.0 Hz, 1H, OH), 4.88–4.84 (m, 1H, H-2″), 4.24 (d, *J* = 13.8 Hz, 1H, SCH), 4.19 (d, *J* = 13.8 Hz, 1H, SCH), 4.11–4.06 (m, 1H, H-3″), 3.81–3.78 (m, 1H, H-4″), 3.63–3.57 (m, 1H, H-5″), 3.50–3.43 (m, 1H, H-5″), 2.33 (s, 3H, CH_3_); ^13^C-NMR (DMSO-*d*_6_, 100 MHz) δ 160.31 (C=O), 155.64 (C=O), 153.25, 152.53, 151.08, 141.71, 140.95, 134.00, 132.61, 127.96, 124.08, 118.61, 117.38, 115.87, 88.29, 85.77, 70.60, 62.03, 32.65 (SCH_2_), 20.23 (CH_3_); IR (ATR) 3301 (br, OH + NH), 1720 (m, C=O), 1644 (s, C=O), 1566 (m), 1081 (s) cm^−1^; MS (FAB^+^) *m*/*z* 487 (M^+^, 1), 185 (9), 97 (31), 55 (100); HRMS (FAB) calcd for (C_21_H_21_N_5_O_7_S)^+^: 487.1162, found: 487.1162.

## 5. Conclusions

Four new compound sets containing 20 imidazole–coumarin conjugates were established. Experimental results indicate that five among the 20 conjugates significantly inhibited HCV subgenomic replicon replication in the Huh 5-2 cell lines. In particular, the series of 1*H*-imidazole conjugates (*i.e.*, **3b**, **3d**, and **3e**) exhibited the most appealing results, with EC_50_ values of 7.2, 5.1, and 8.4 μM together with SI values of 12, 15, and 21, respectively. The imidazole ring can be *N*-alkylated or ribosylated as well as replaced by a benzimidazole, inosine, or guanosine. Nevertheless, the parent imidazole conjugates with an N–H proton generally offers a higher SI value than those without such a proton. Moreover, incorporation of substituents into the coumarin rings can increase both potency and selectivity of the conjugates. These guidelines provide a valuable reference for medicinal scientists attempting to optimize the anti-viral compounds during the drug development stage. The mechanism of action of these conjugated compounds with significant activity towards viral enzymes will be studies in due course.

## Figures and Tables

**Table 1:** Anti-metabolic and antiviral effect of conjugated compounds on HCV 1b subgenomic replicon replication in Huh 5-2 cells.

Compound ^a^	CC_50_^b^ (μM)	EC_50_^c^ (μM)	SI ^d^
**3a**	122	30	4.1
**3b**	83	7.2	12
**3c**	85	9.7	8.8
**3d**	75	5.1	15
**3e**	173	8.4	21
**3f**	49	6.7	7.3
**3g**	75	15	5.1
**7a**	128	59	2.2
**7b**	122	26	4.7
**7c**	85	14	6.3
**7d**	107	19	5.7
**7e**	119	34	3.5
**9a**	109	61	1.8
**9b**	105	73	1.4
**9d**	93	70	1.3
**9e**	102	102	1.0
**9f**	106	106	1.0
**11b**	102	102	1.0
**11e**	99	99	1.0
**11f**	103	103	1.0
**12a** ^e^ [11]	90	27	3.4
**12d** ^e^ [11]	42	4.0	10
**12e** ^e^ [11]	27	10	2.8
coumarin	>500	>150	-
imidazole	>500	>150	-

^a^ Interferon α-2b was used as a (positive) reference compound at 10,000 units/well and reduced the signal in the viral RNA (luciferase) assay to background levels. The values were obtained as the average of triplicate determinations; ^b^ The concentration of a compound with an adverse effect of 50% was observed on the host cell metabolism, as determined by the MTS method; ^c^ The concentration of a compound at which virus replication was inhibited by 50% was observed, as determined by real-time quantitative RT-PCR; ^d^ Selectivity index (ratio of CC_50_ to EC_50_); ^e^ Reference known compounds published in [11].

## Supplementary Materials

Supplementary materials can be accessed at: http://www.mdpi.com/1420-3049/21/2/228/s1.
