# Supplementary material for: Synthesis and Structure-Activity Relationships of Imidazole-Coumarin Conjugates against Hepatitis C Virus
Source: Molecules. 2016 Feb 18;21(2):228. doi: 10.3390/molecules21020228 (PMC6273635; doi:10.3390/molecules21020228)
Supplement: Supplementary file 1 [file molecules-21-00228-s001.pdf]

Synthesis and Structure-Activity Relationship of Imidazole-Coumarin Conjugates against Hepatitis C Virus

Shwu-Chen Tsay, Shu-Yu Lin, Wen-Chieh Huang, Ming-Hua Hsu, Kuo Chu Hwang, Chun-Cheng Lin, Jia-Cherng Horng, I-Chia Chen, Jih Ru Hwu, Fa-Kuen Shieh, Pieter Leyssen and Johan Neyts

Table S1. Compound Purity by HPLC.

Compound	Gradient A/B	Purity (%)	t_R (min)
**3a**	20:80	98.5	7.52
**3b**	20:80	99.0	8.02
**3c**	20:80	98.6	9.58
**3d**	20:80	98.8	8.55
**3e**	20:80	99.6	7.83
**3f**	20:80	99.4	8.61
**3g**	20:80	99.8	10.44
**7a**	20:80	98.0	6.37
**7b**	20:80	98.1	7.16
**7c**	20:80	99.0	8.19
**7d**	20:80	98.9	8.47
**7e**	20:80	99.3	6.85
**9a**	20:80	98.2	6.68
**9b**	20:80	98.7	6.87
**9d**	20:80	99.2	7.12
**9e**	20:80	98.6	7.45
**9f**	20:80	98.8	7.36
**11b**	20:80	98.3	7.59
**11e**	20:80	98.4	7.41
**11f**	20:80	99.3	7.37

Mobile phase: solvent A, acetonitrile; solvent B, H2O; Detection Time: totally 20 min; Detection: l254 nm; Column: Thermo 5 mm Hypersil ODS (250 × 4.6 mm D.I.); Flow rate: 0.80 mL/min.

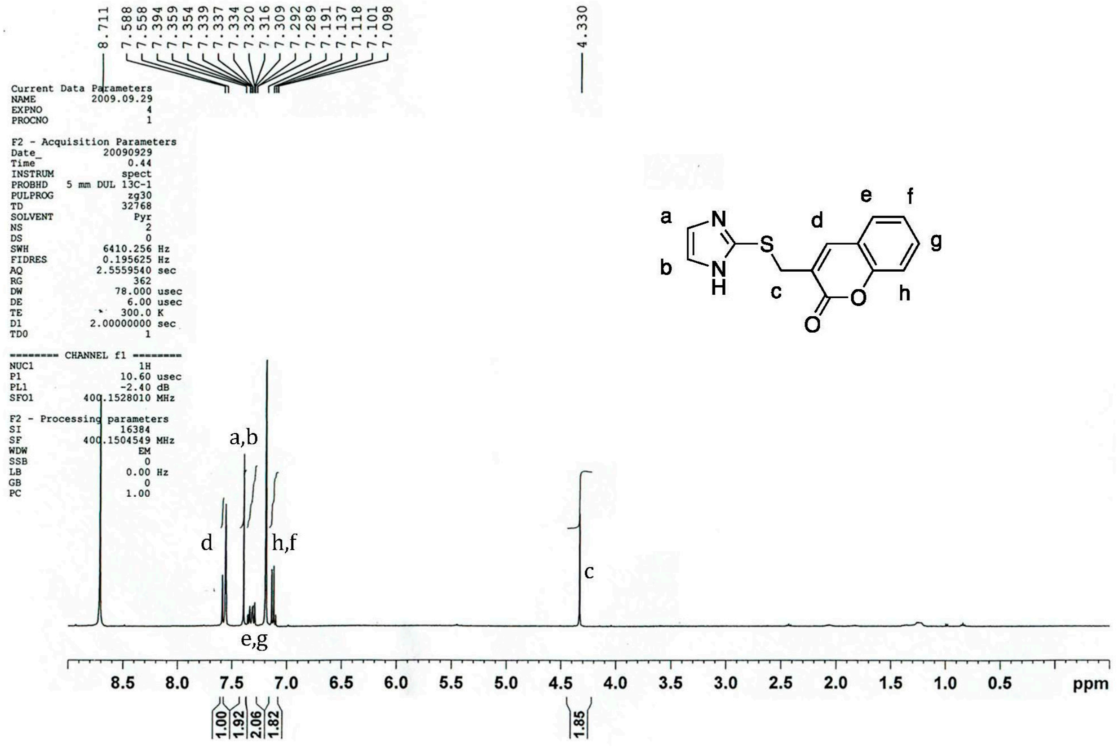

Figure S1. ^{1}H NMR spectrum of compound **3a**.

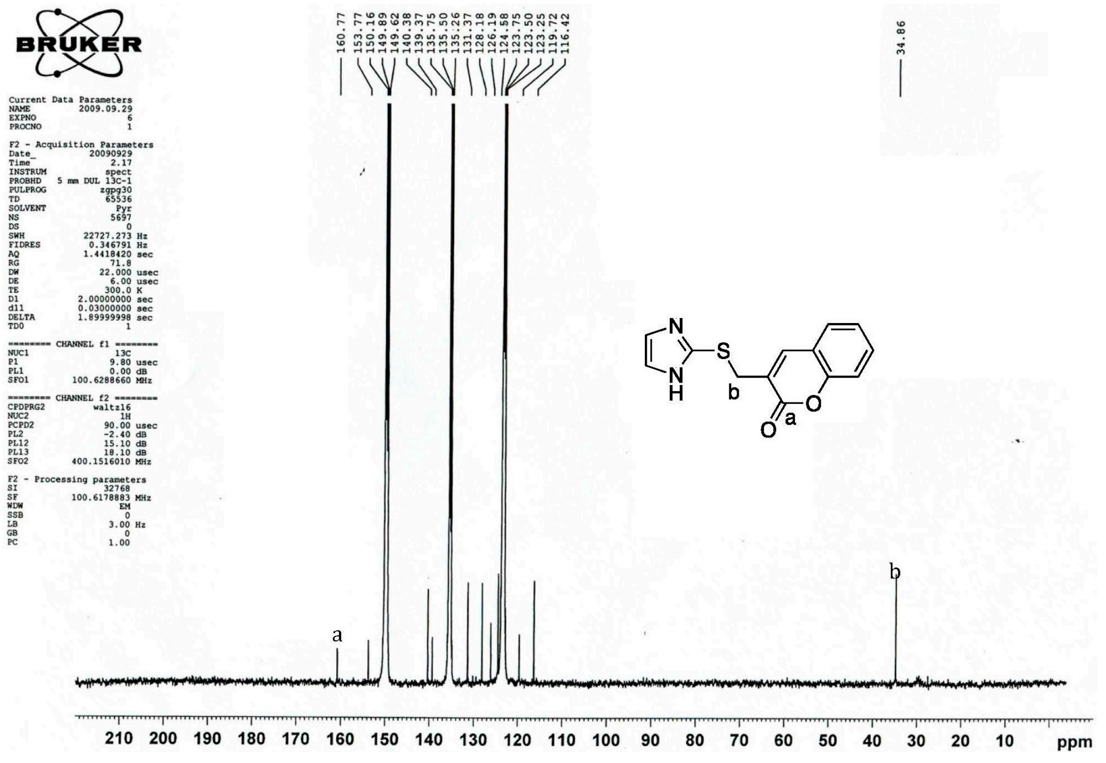

Figure S2. ^{13}C NMR spectrum of compound **3a**.

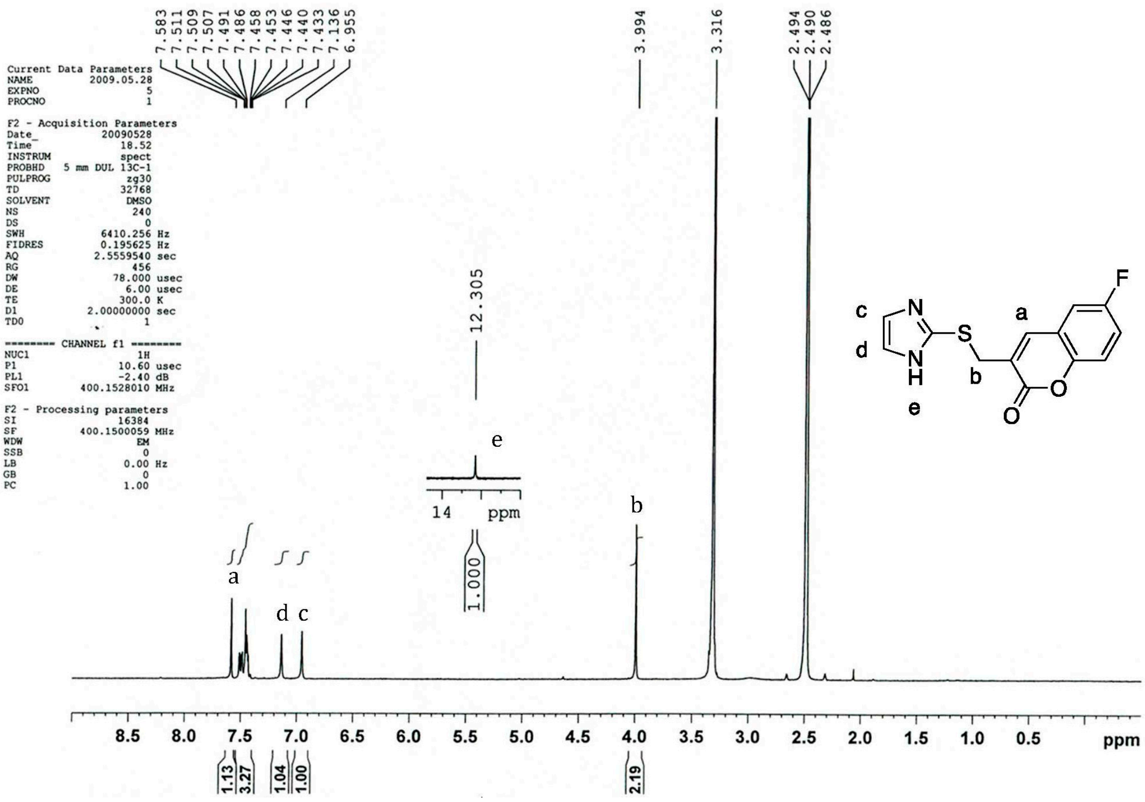

Figure S3. ^{1}H NMR spectrum of compound **3b**.

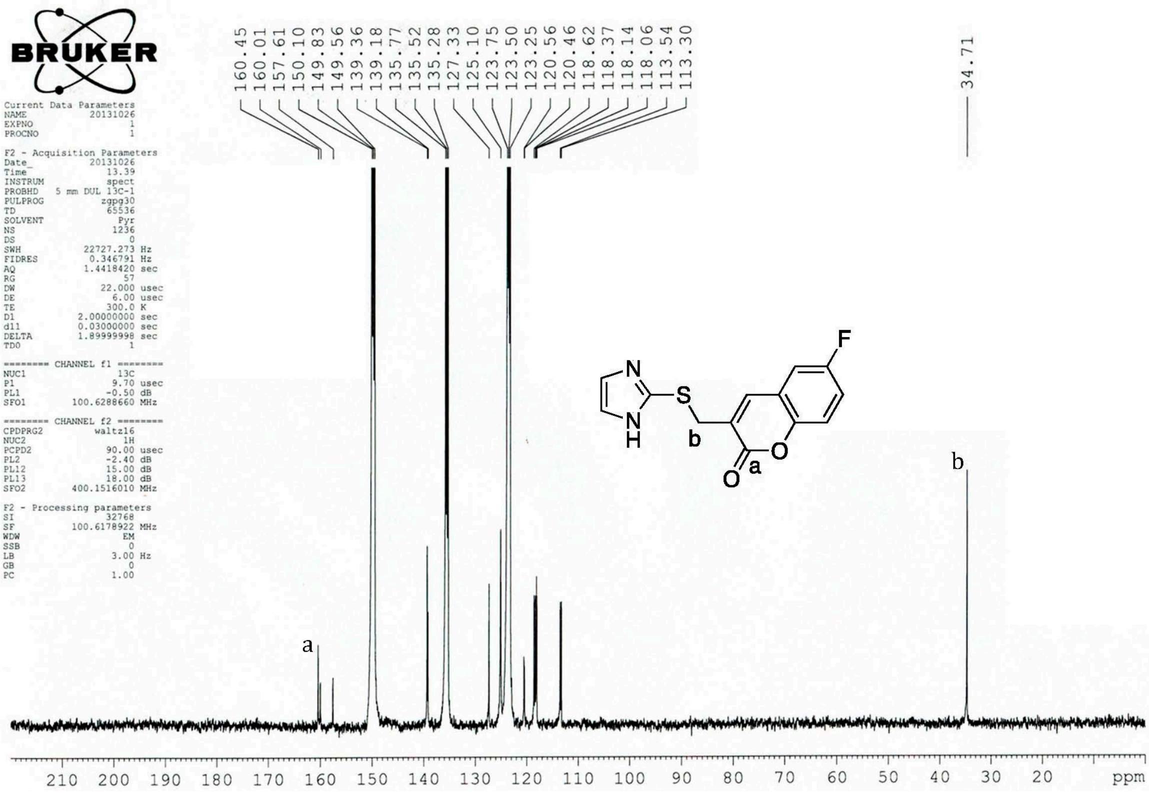

Figure S4. ^{13}C NMR spectrum of compound **3b**.

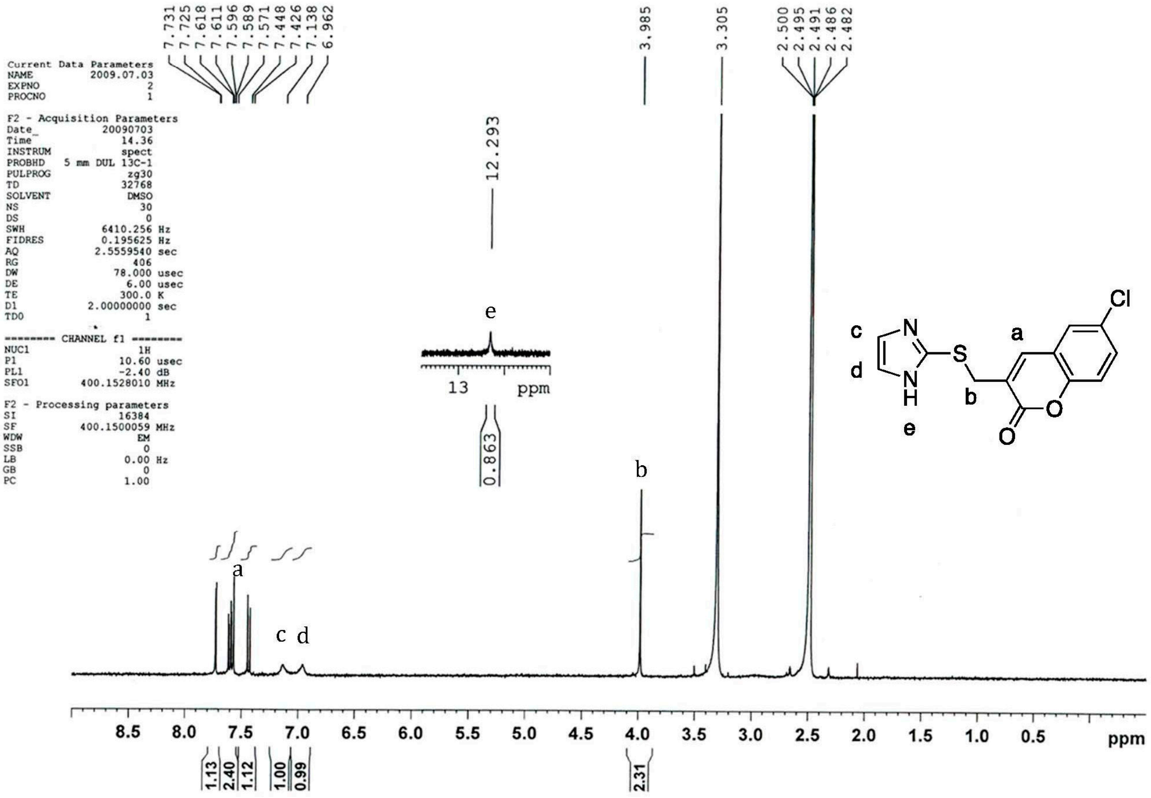

Figure S5. ^{1}H NMR spectrum of compound **3c**.

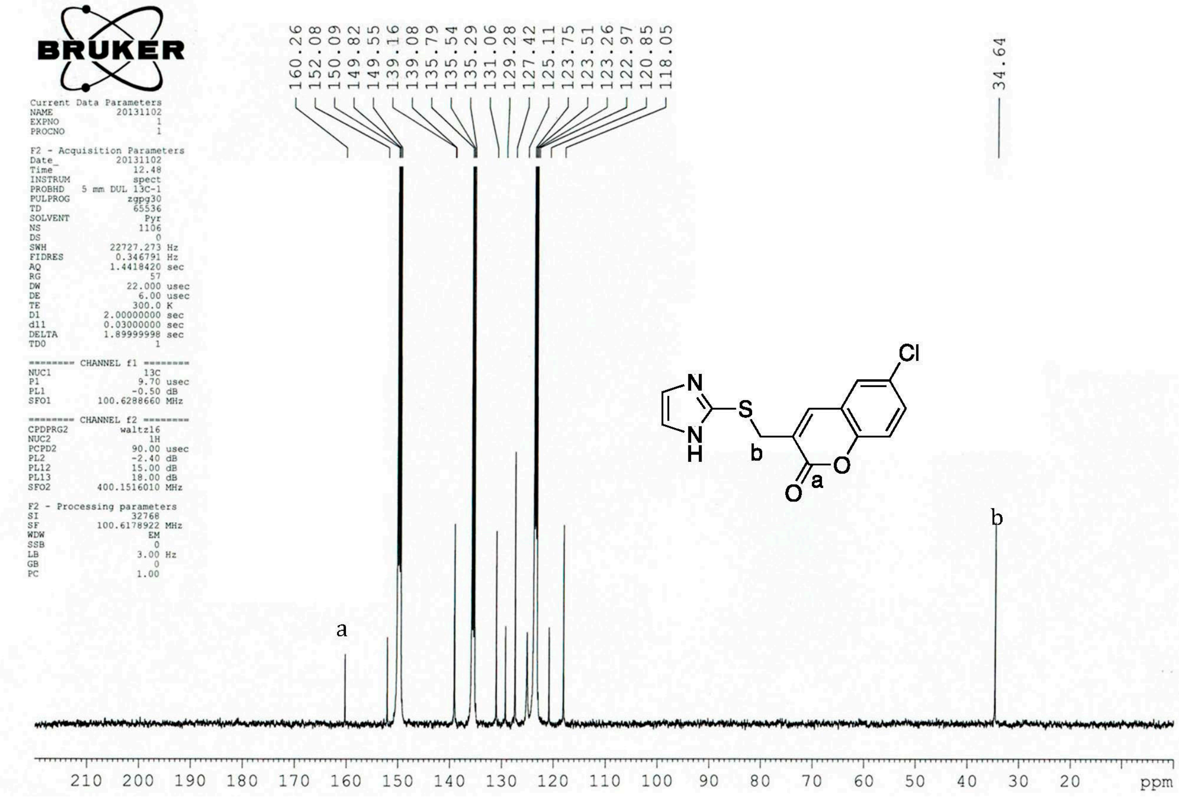

Figure S6. ^{13}C NMR spectrum of compound **3c**.

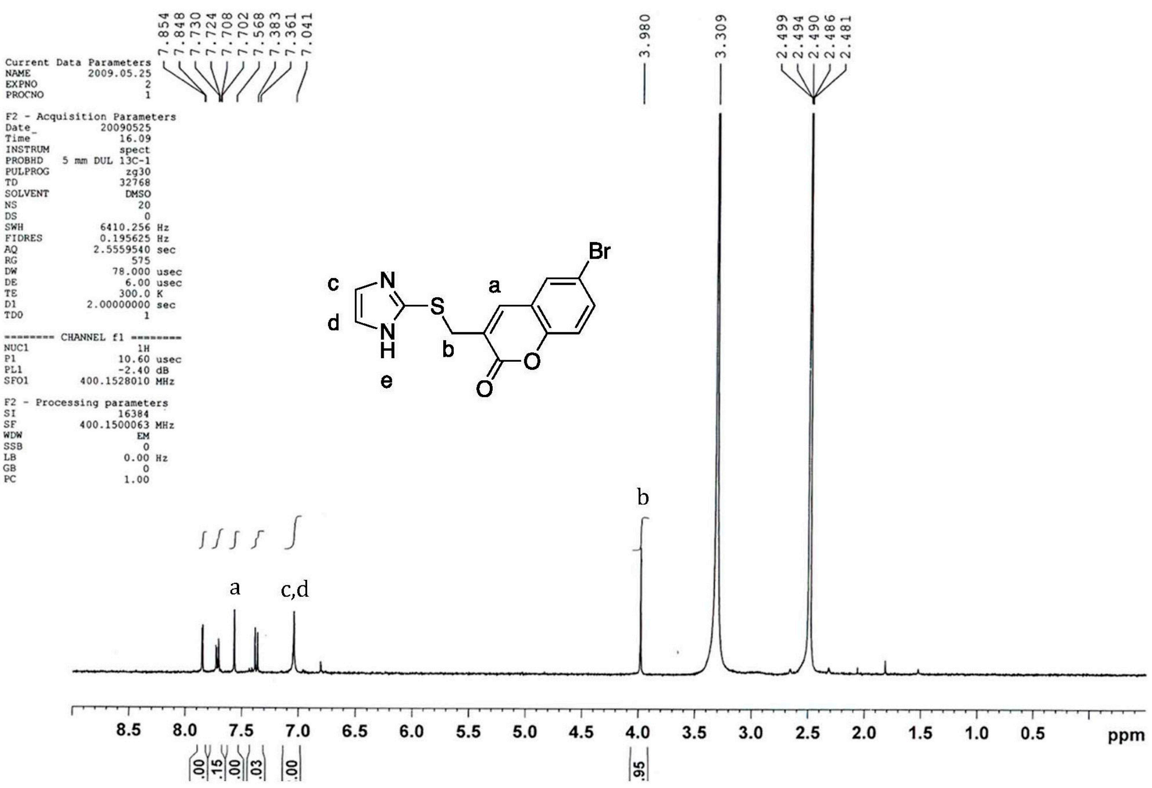

Figure S7. ^{1}H NMR spectrum of compound **3d**.

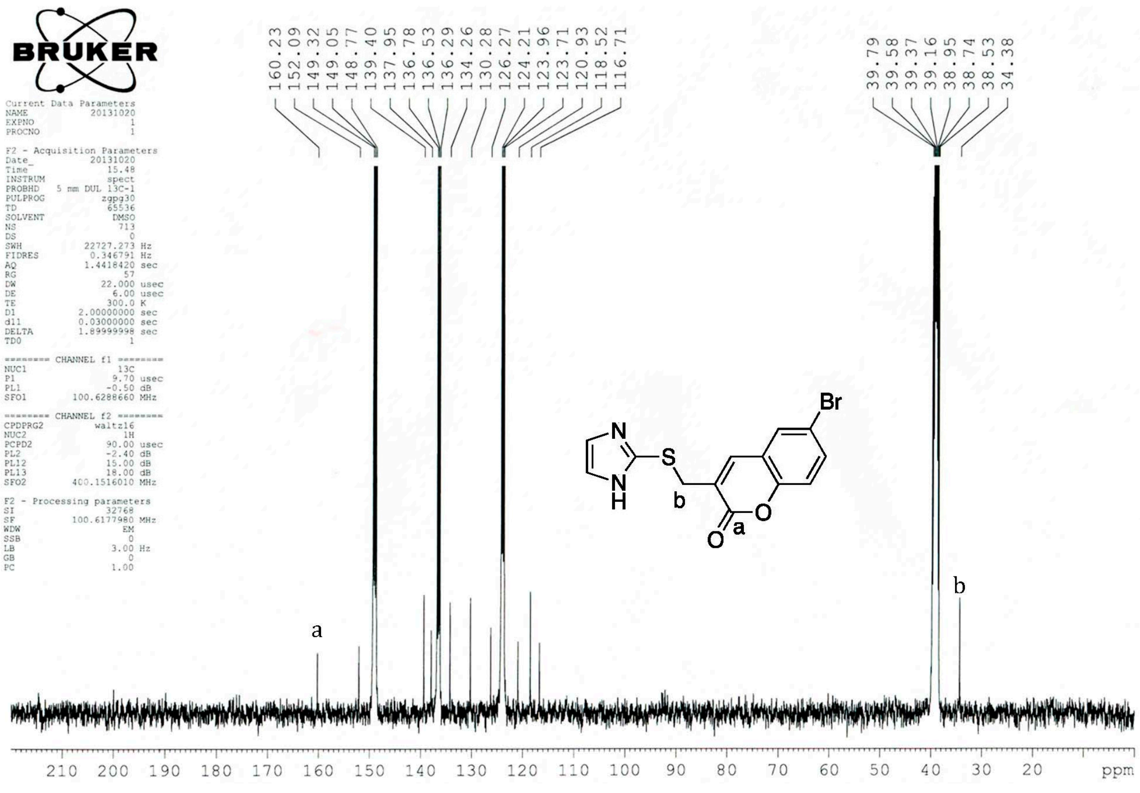

Figure S8. ^{13}C NMR spectrum of compound **3d**.

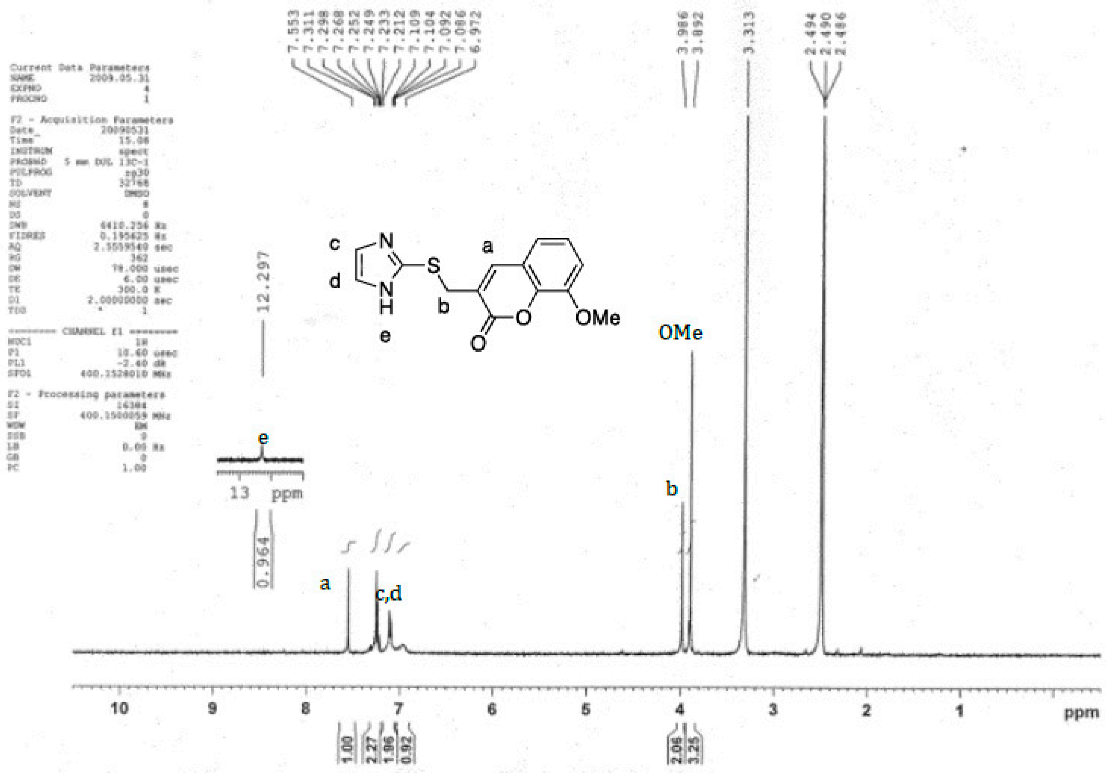

Figure S9. ^{1}H NMR spectrum of compound **3e**.

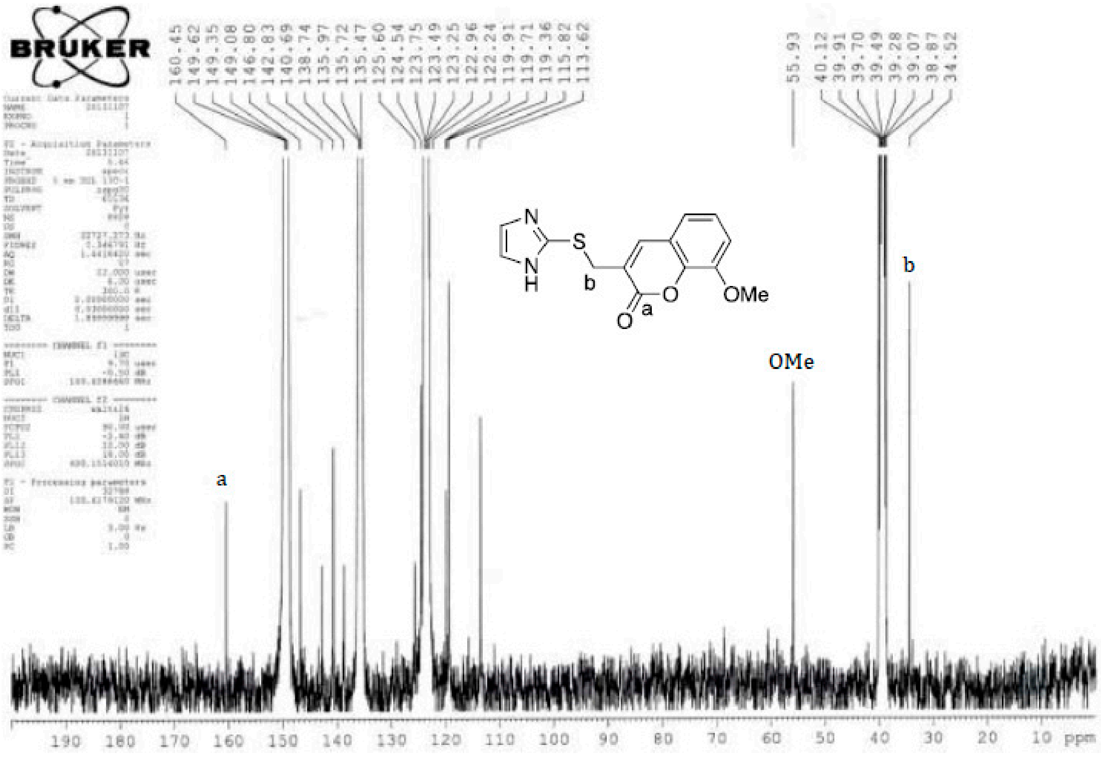

Figure S10. ^{13}C NMR spectrum of compound **3e**.

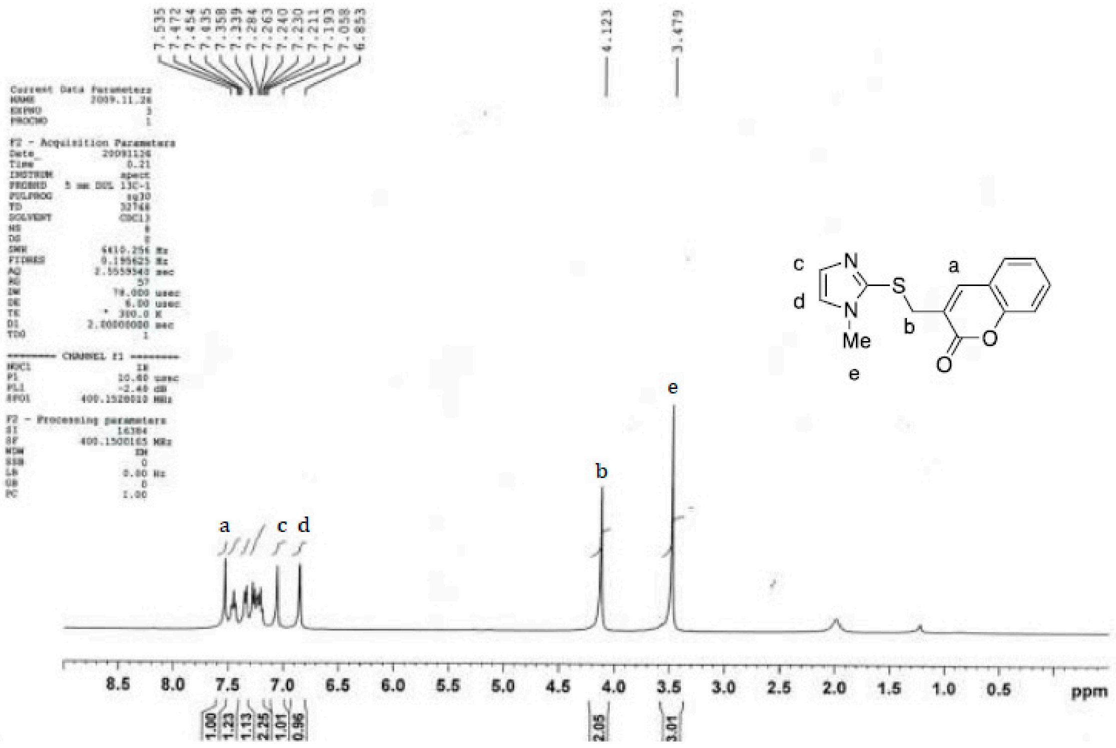

Figure S11. ^{1}H NMR spectrum of compound **3f**.

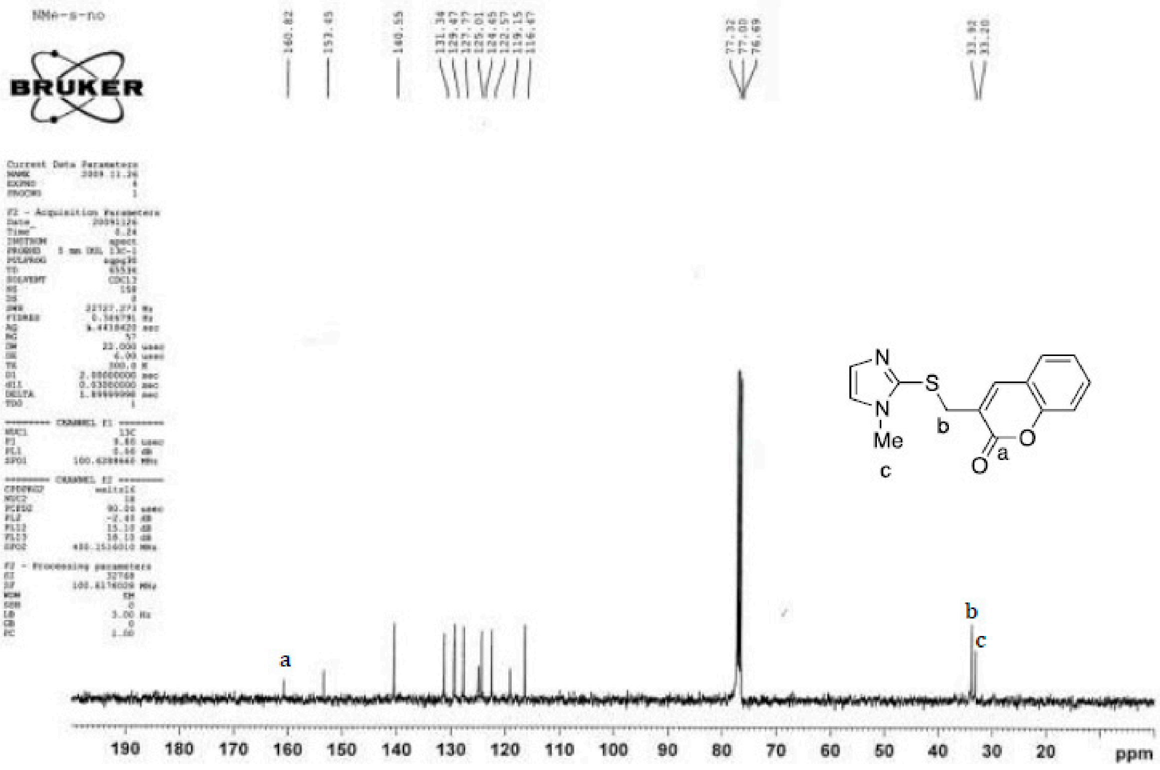

Figure S12. ^{13}C NMR spectrum of compound **3f**.

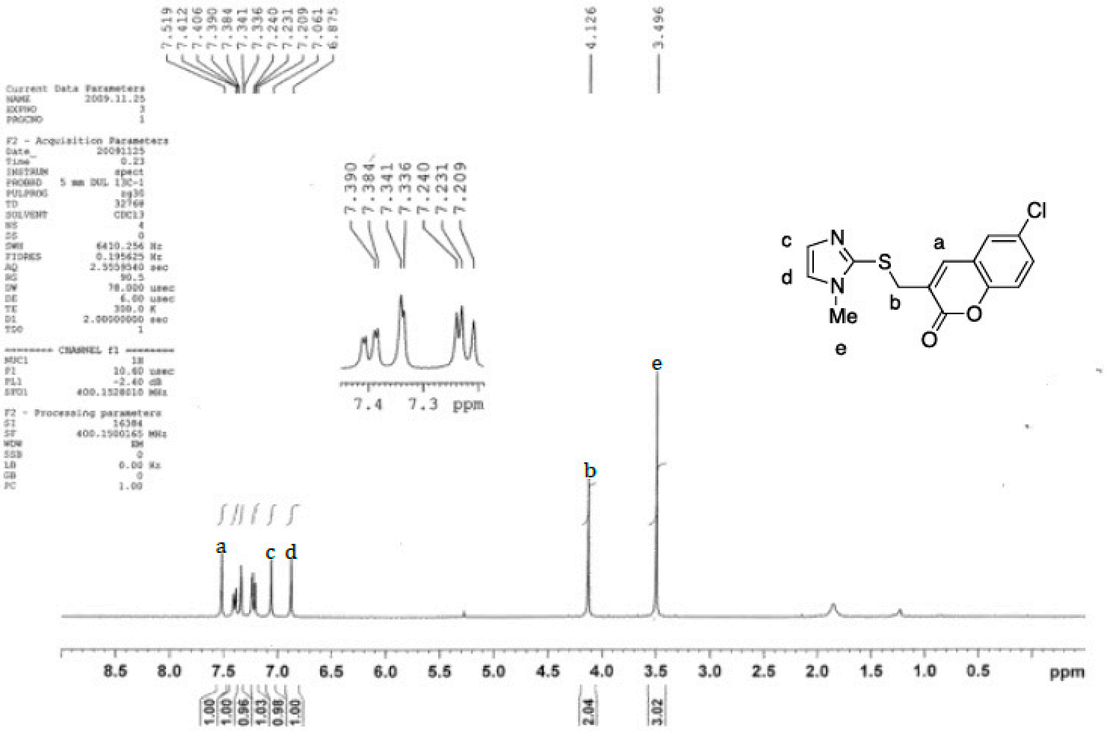

Figure S13. ^{1}H NMR spectrum of compound **3g**.

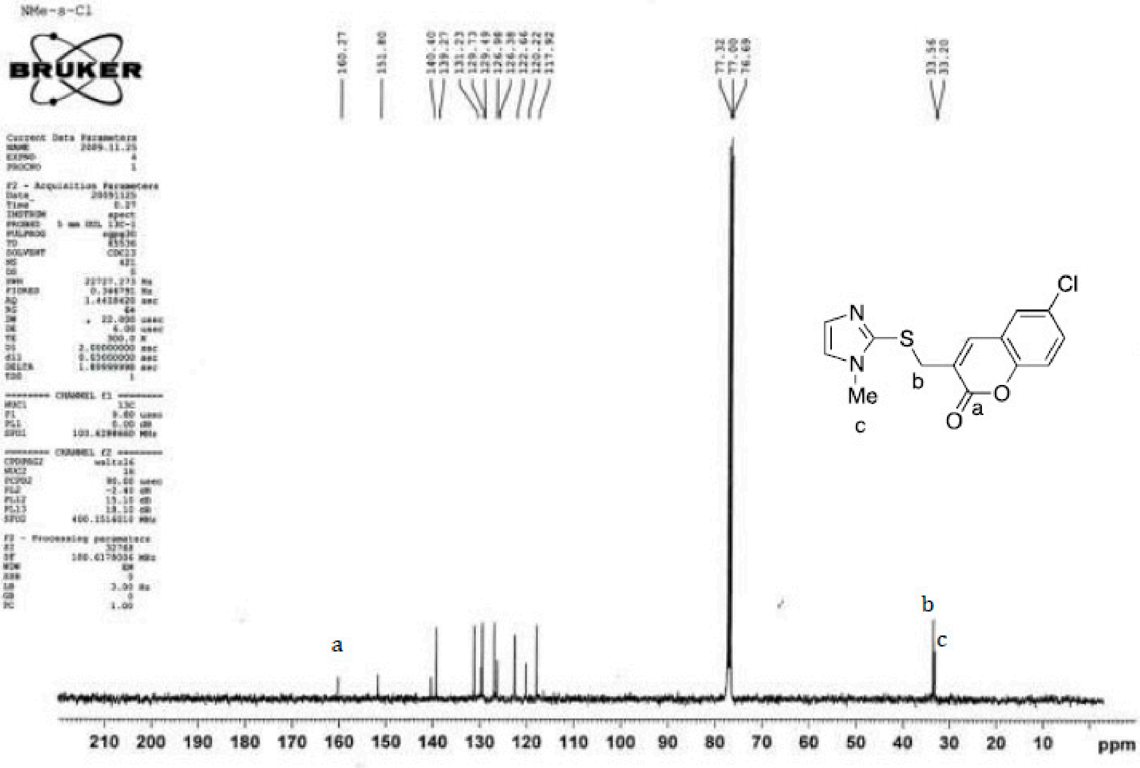

Figure S14. ^{13}C NMR spectrum of compound **3g**.

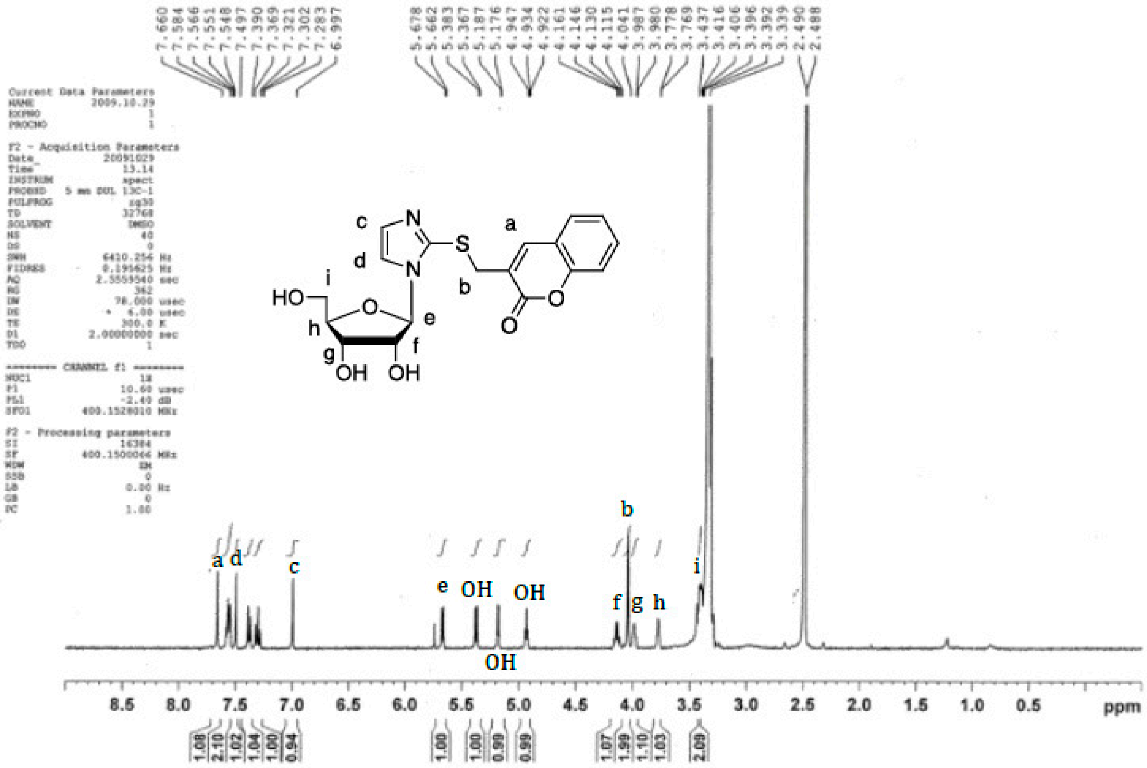

Figure S15. ^{1}H NMR spectrum of compound **7a**.

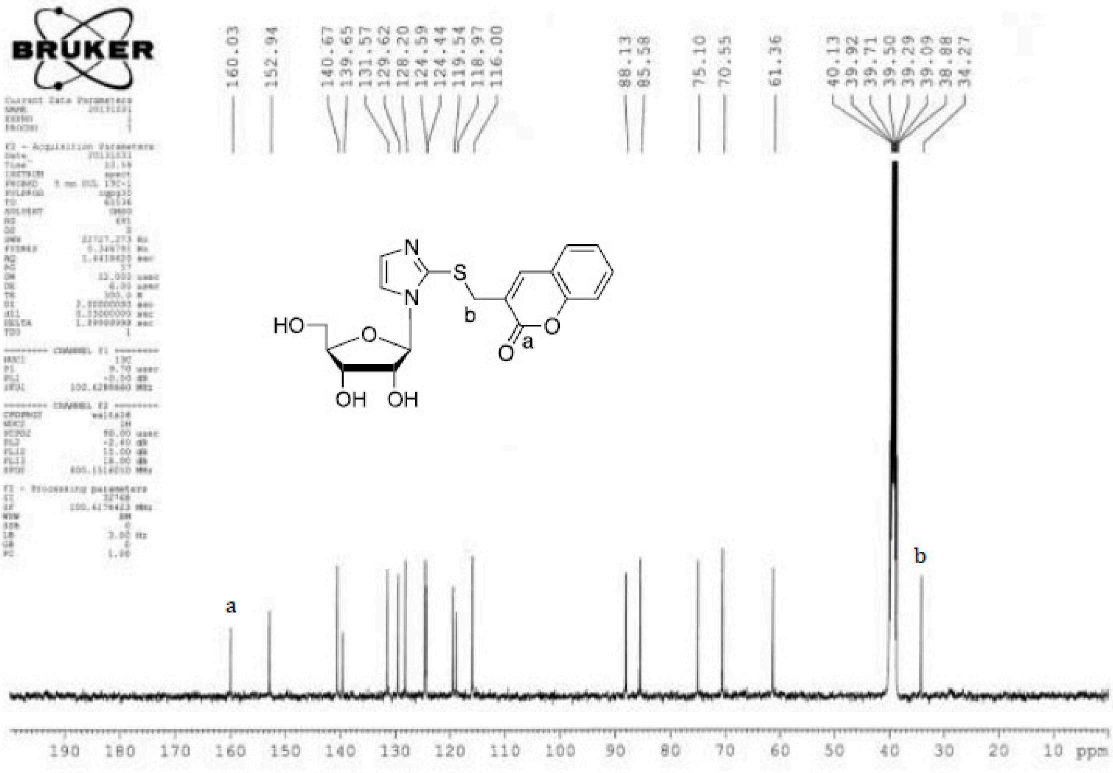

Figure S16. ^{13}C NMR spectrum of compound **7a**.

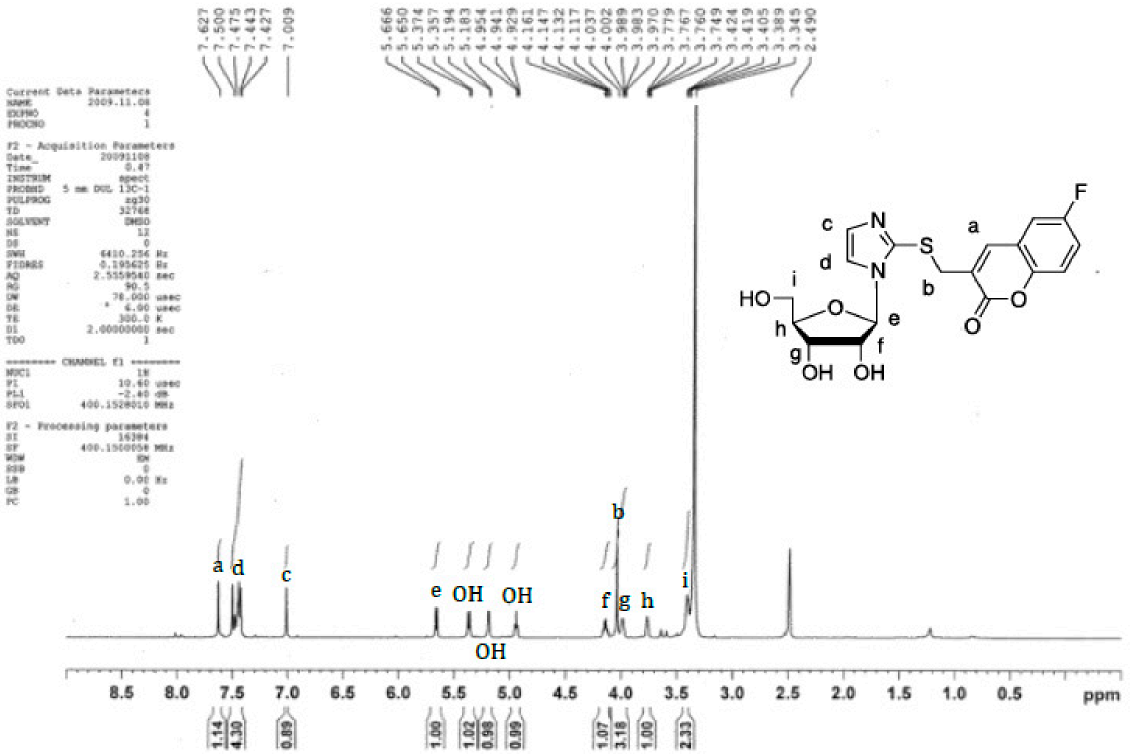

Figure S17. ^{1}H NMR spectrum of compound **7b**.

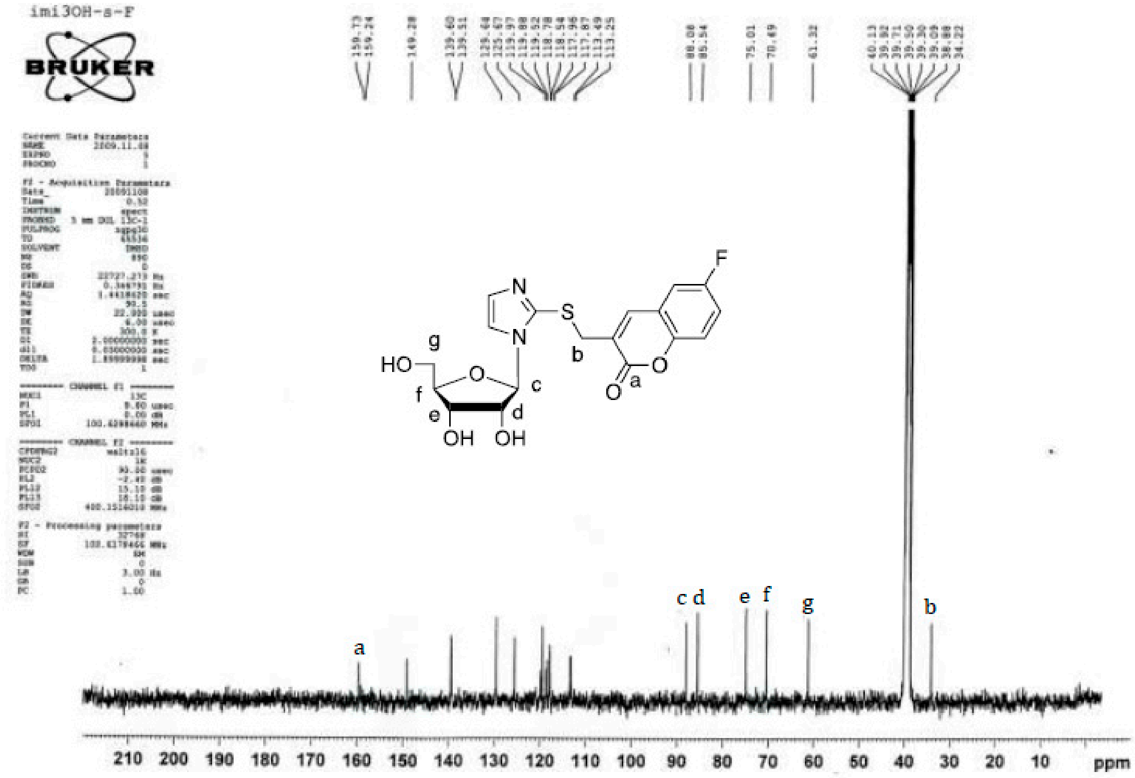

Figure S18. ^{13}C NMR spectrum of compound **7b**.

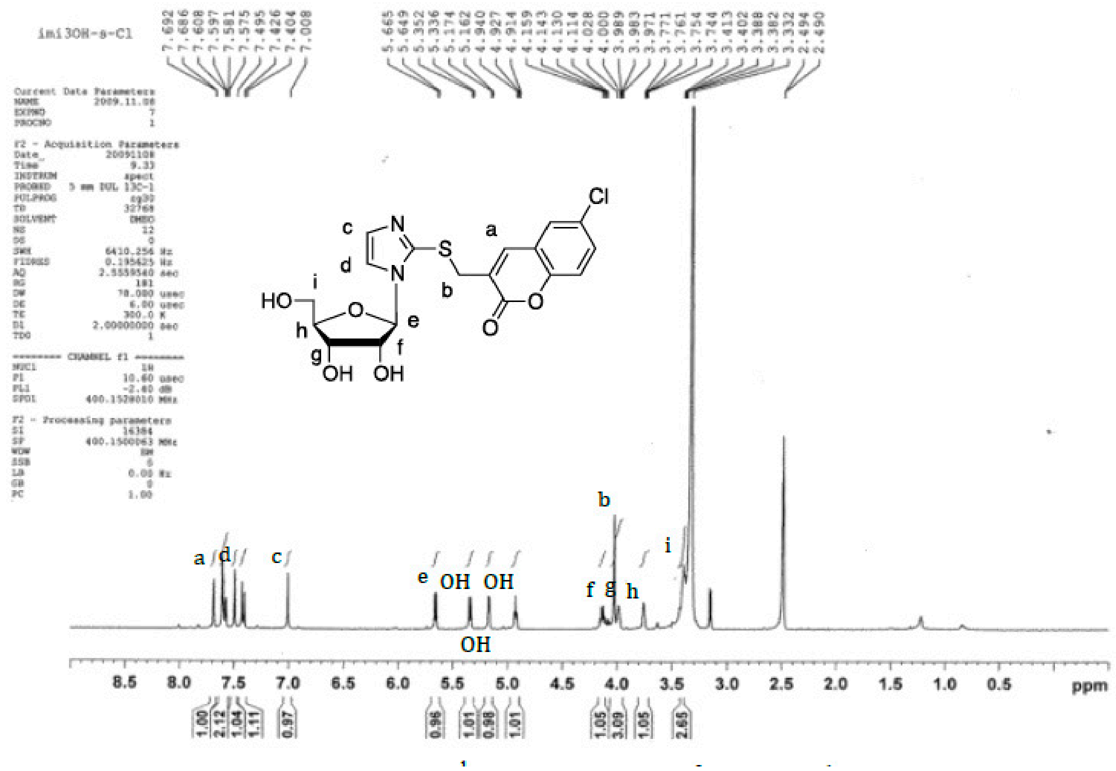

Figure S19. ^{1}H NMR spectrum of compound **7c**.

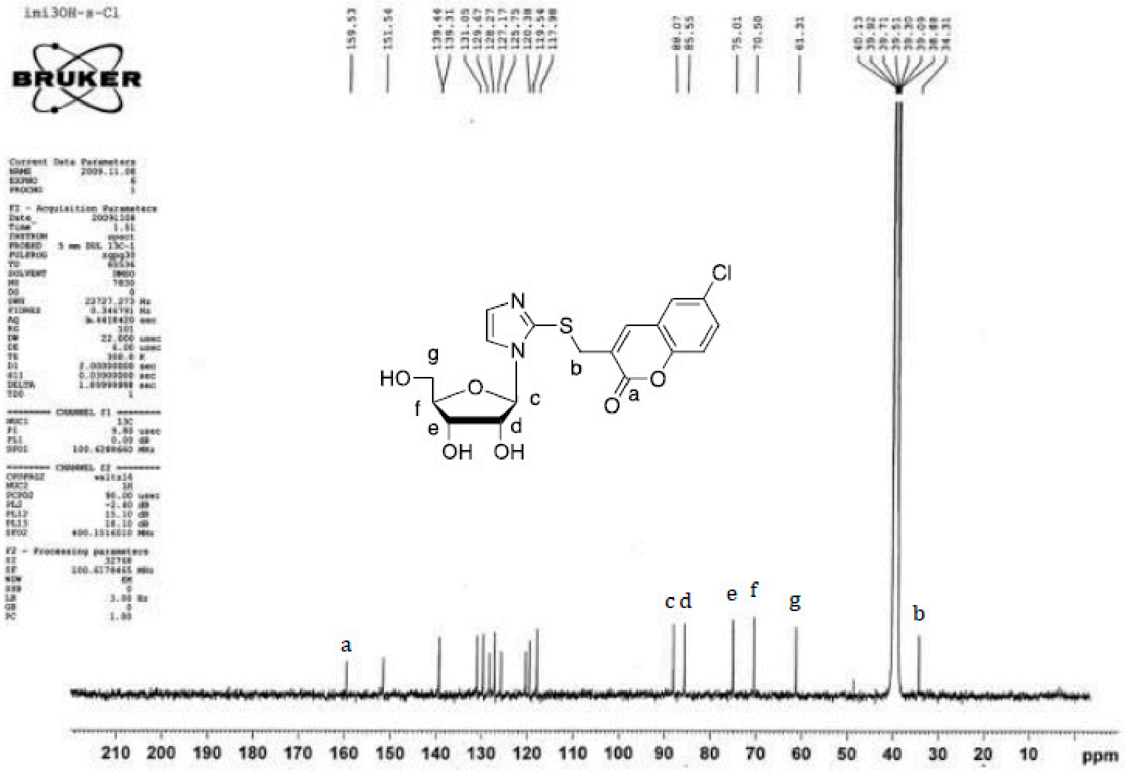

Figure S20. ^{13}C NMR spectrum of compound **7c**.

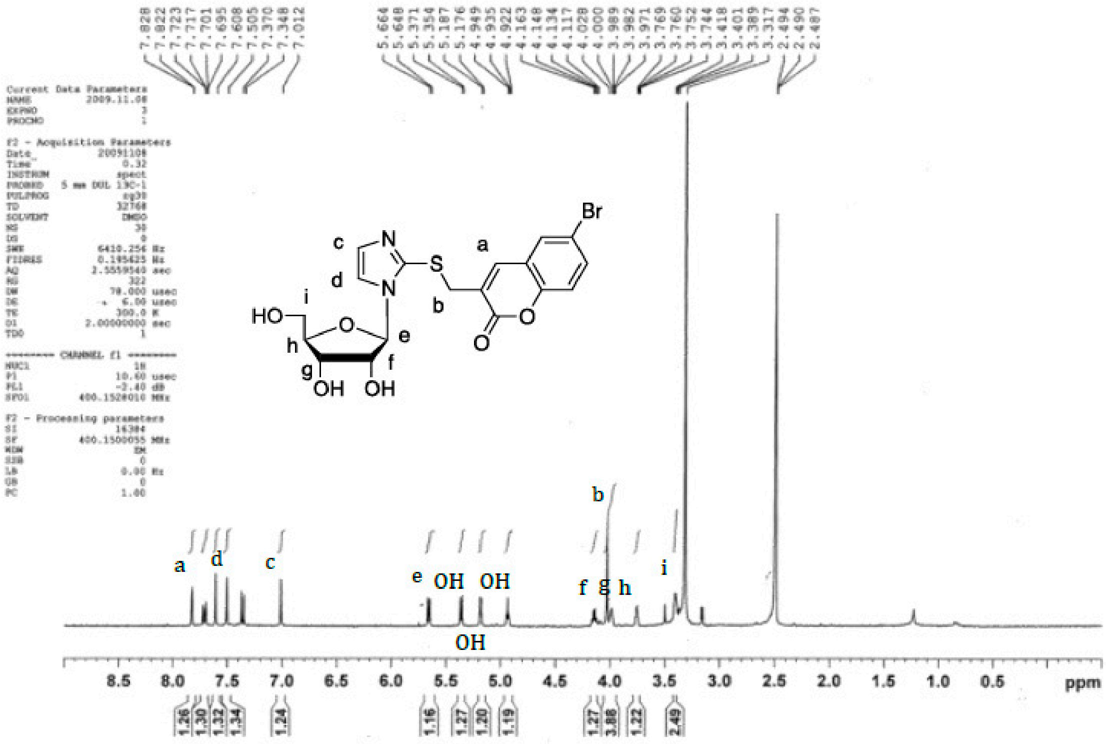

Figure S21. ^{1}H NMR spectrum of compound **7d**.

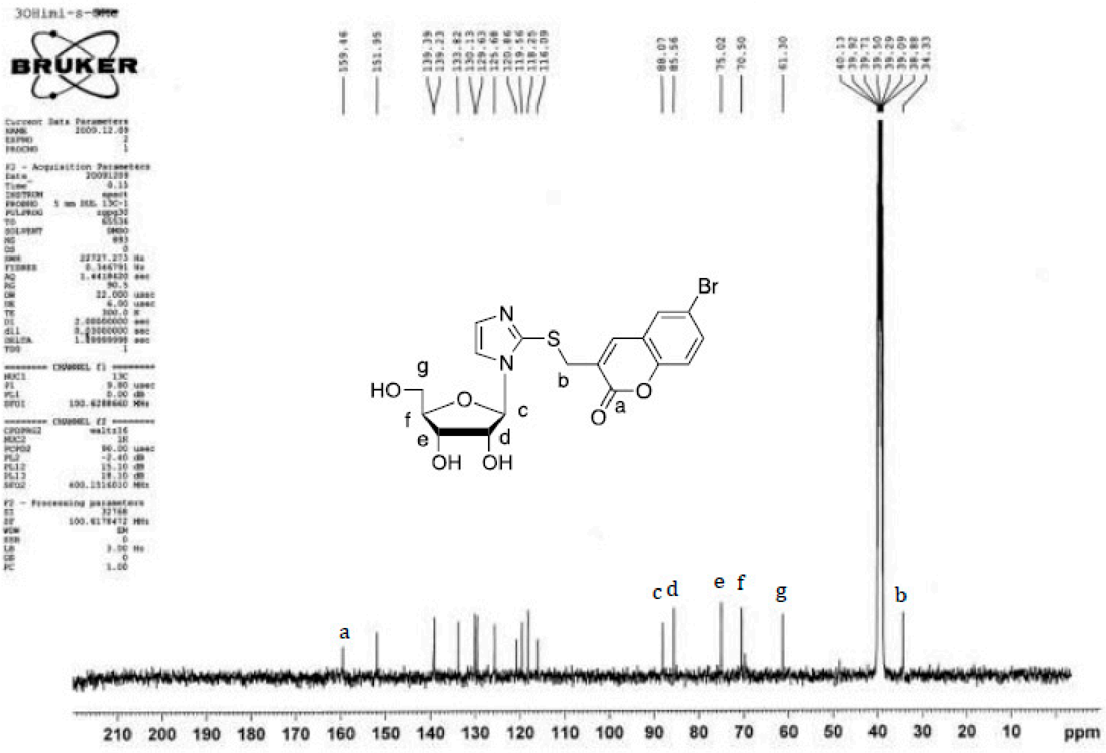

Figure S22. ^{13}C NMR spectrum of compound **7d**.

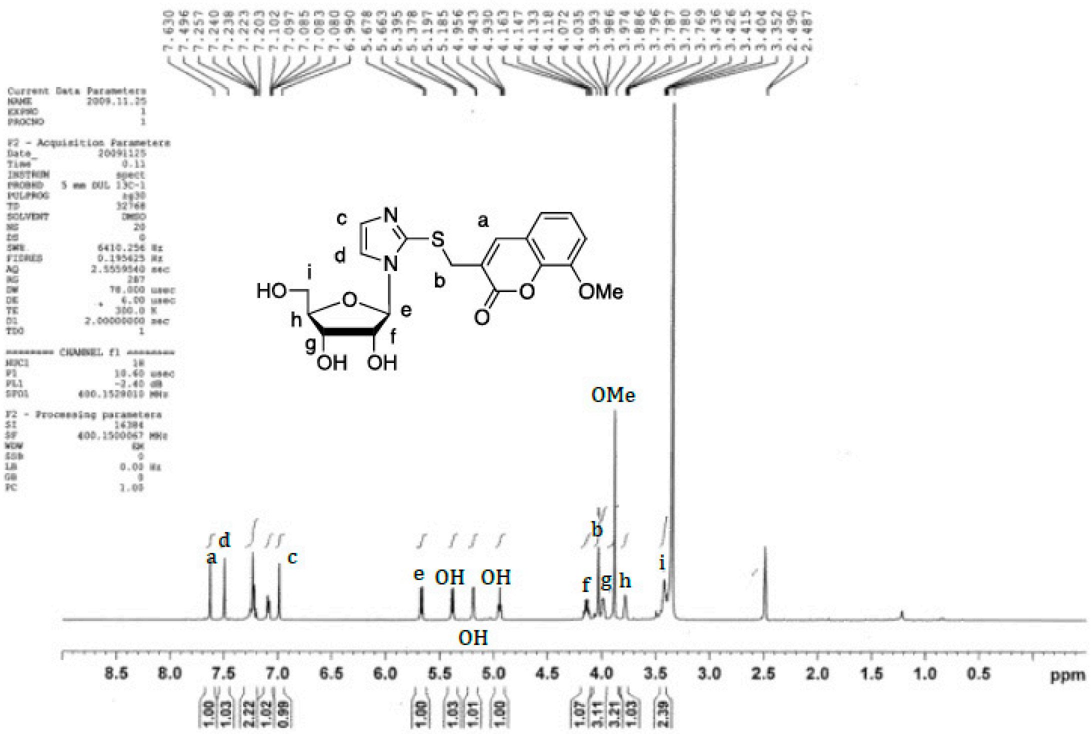

Figure S23. ^{1}H NMR spectrum of compound **7e**.

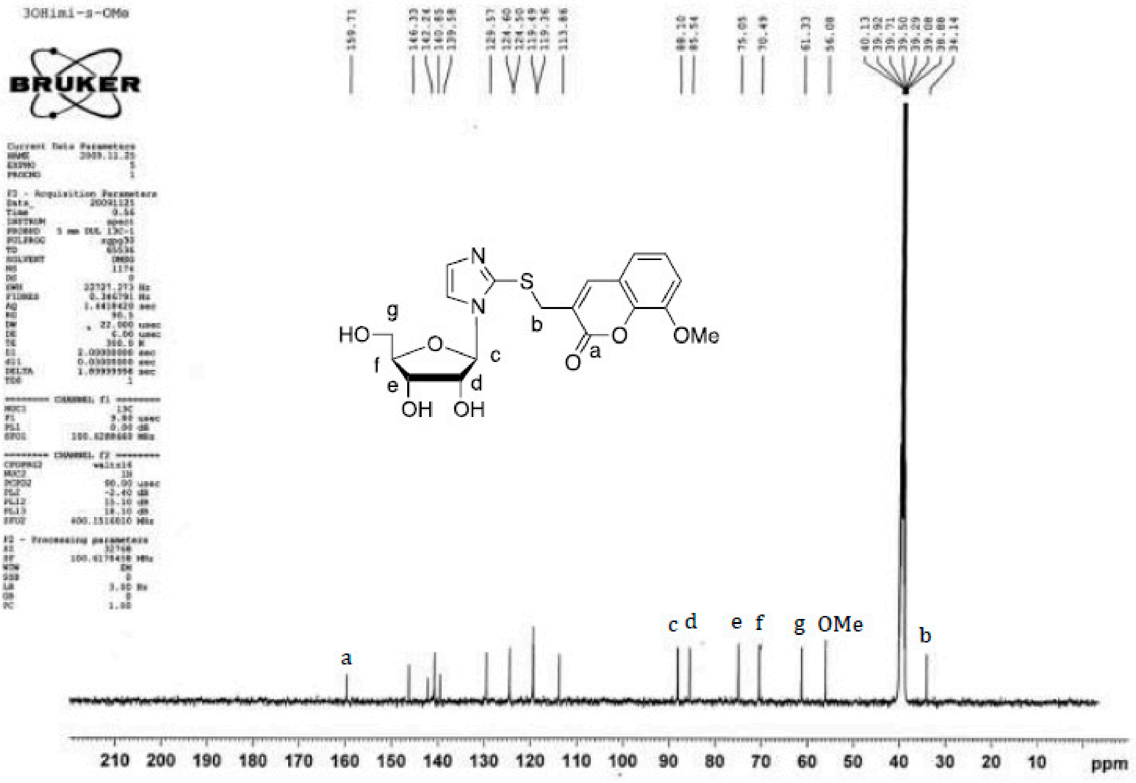

Figure S24. ^{13}C NMR spectrum of compound **7e**.

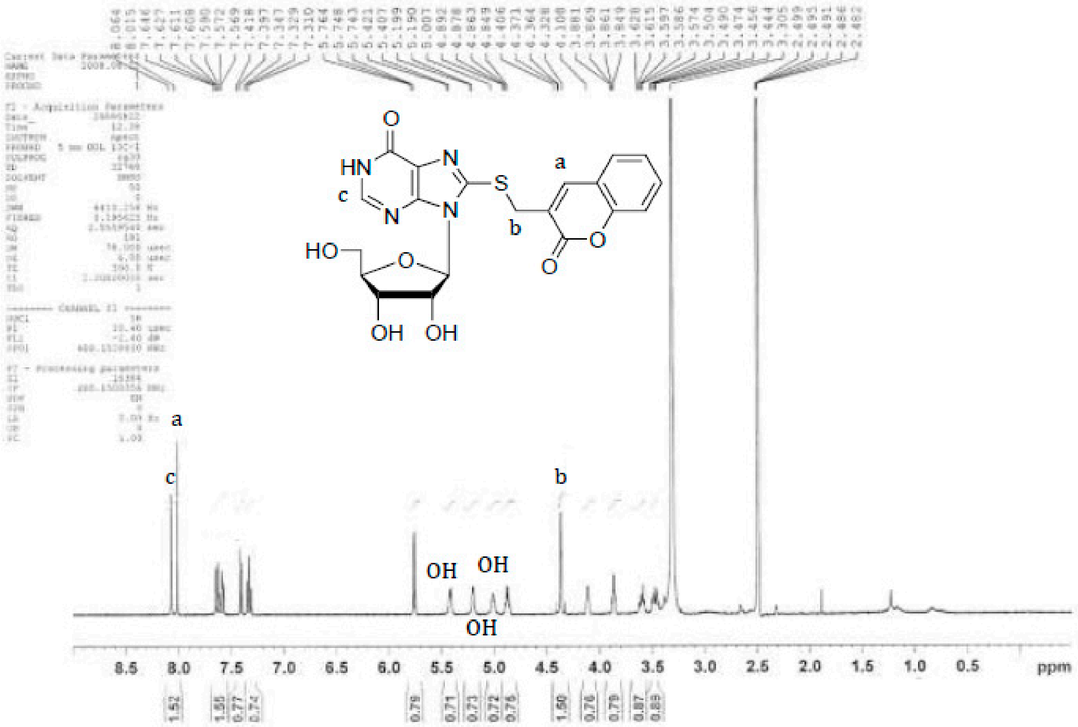

Figure S25. ^{1}H NMR spectrum of compound **9a**.

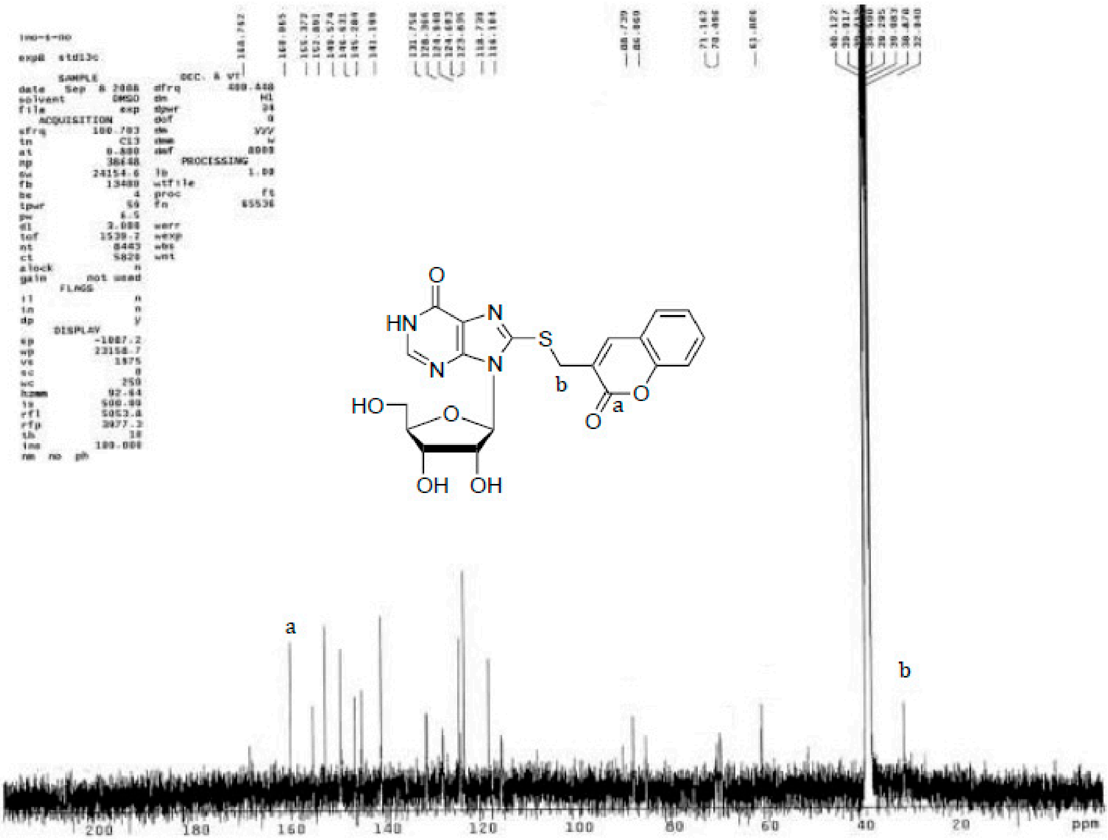

Figure S26. ^{13}C NMR spectrum of compound **9a**.

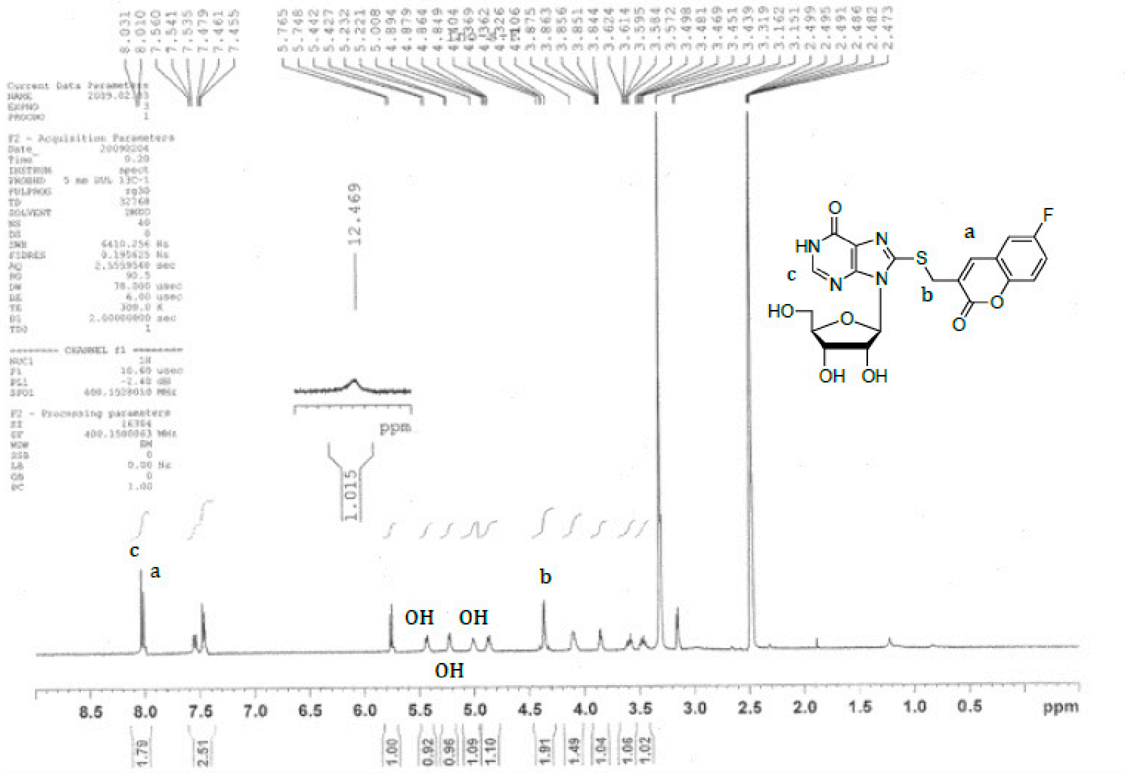

Figure S27. ^{1}H NMR spectrum of compound **9b**.

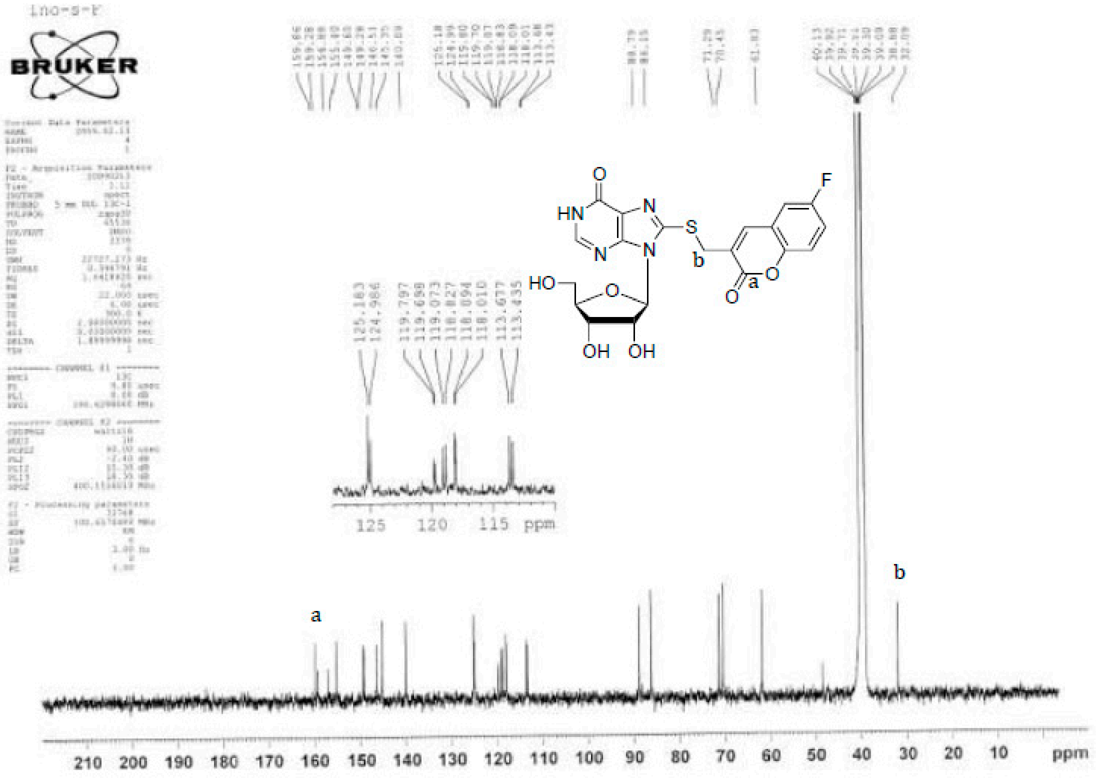

Figure S28. ^{13}C NMR spectrum of compound **9b**.

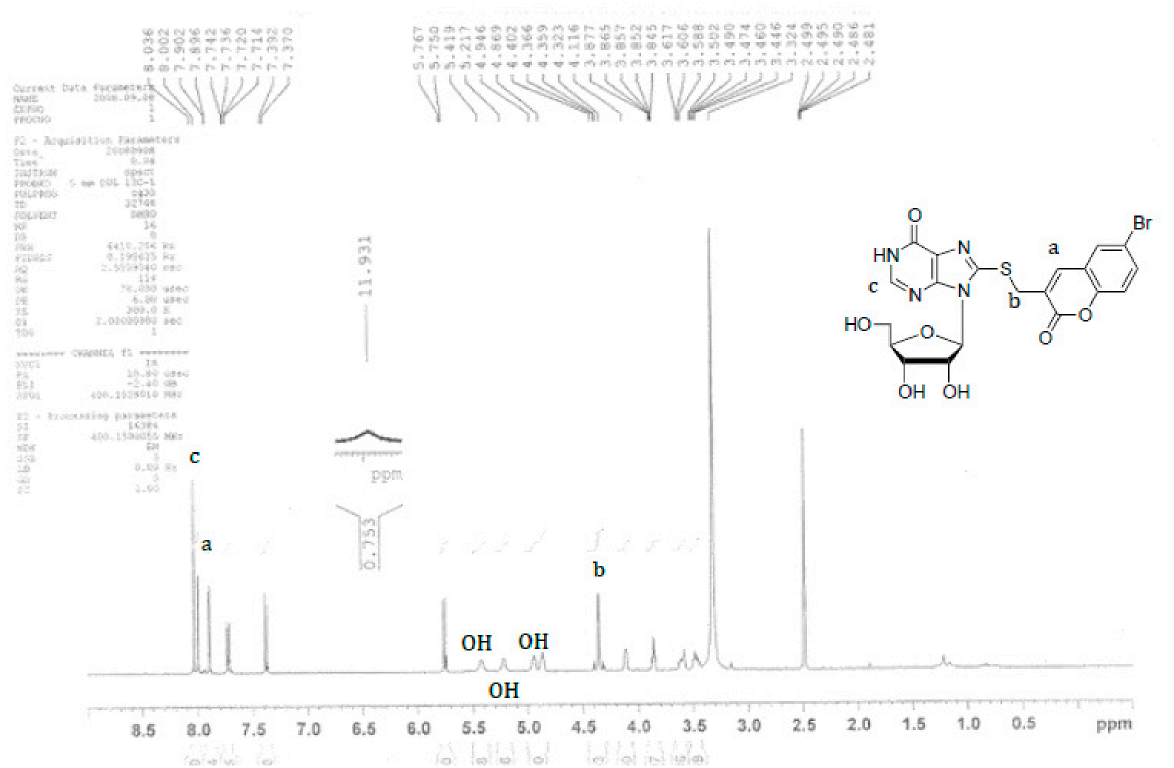

Figure S29. ^{1}H NMR spectrum of compound **9d**.

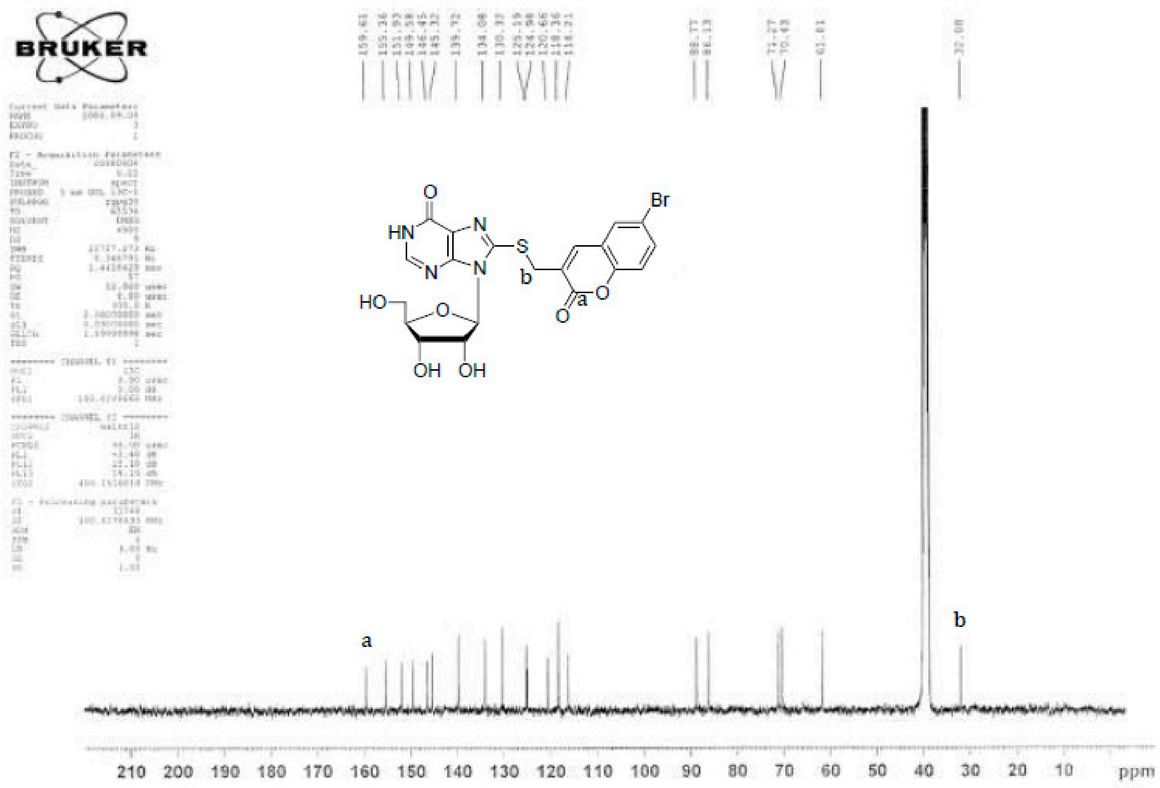

Figure S30. ^{13}C NMR spectrum of compound **9d**.

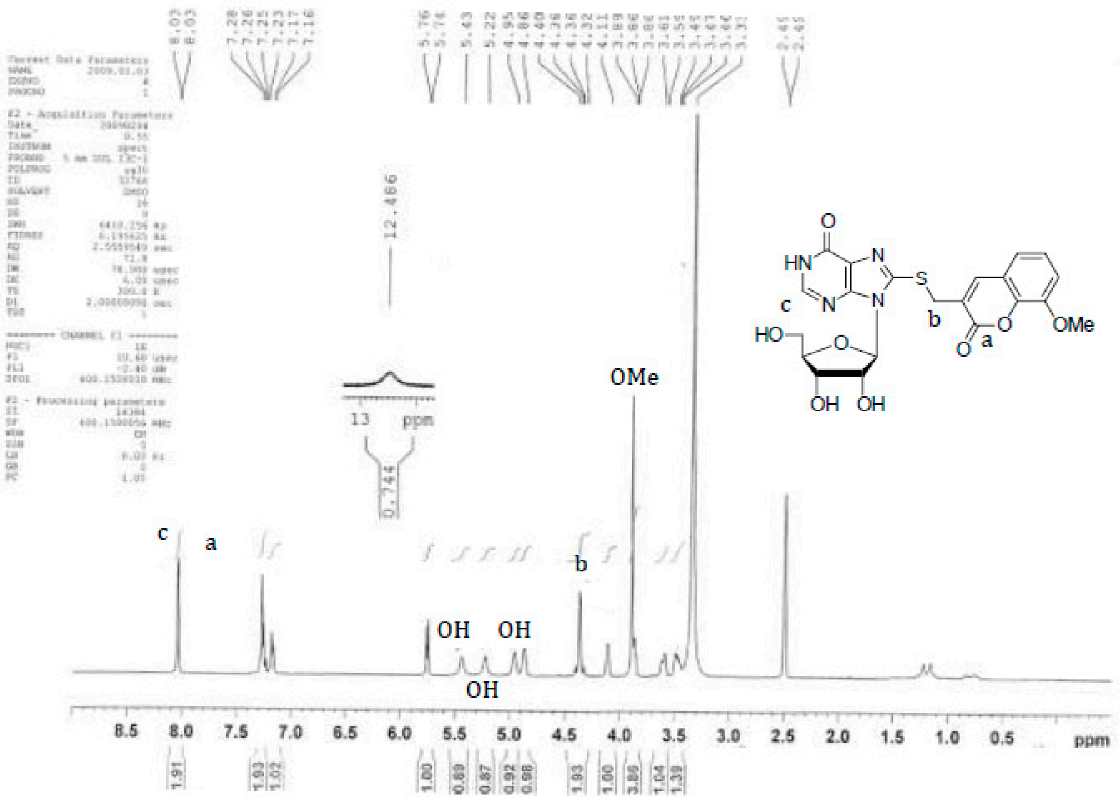

Figure S31. ^{1}H NMR spectrum of compound **9e**.

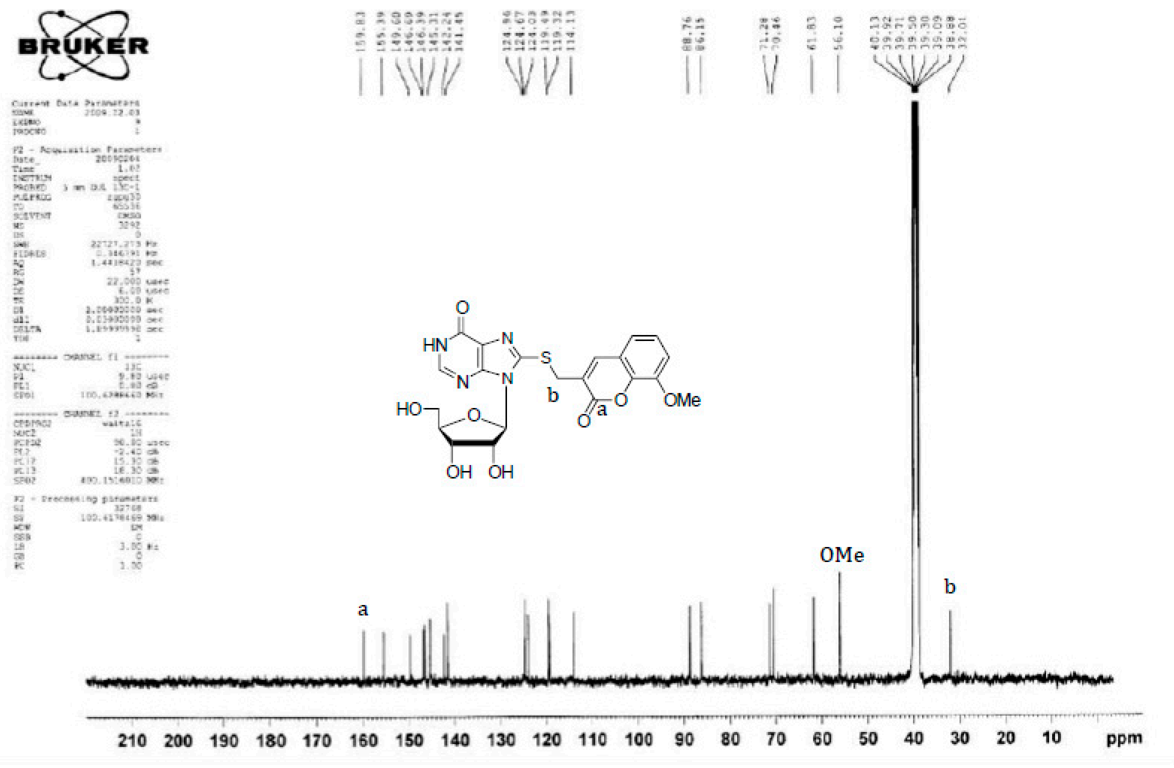

Figure S32. ^{13}C NMR spectrum of compound **9e**.

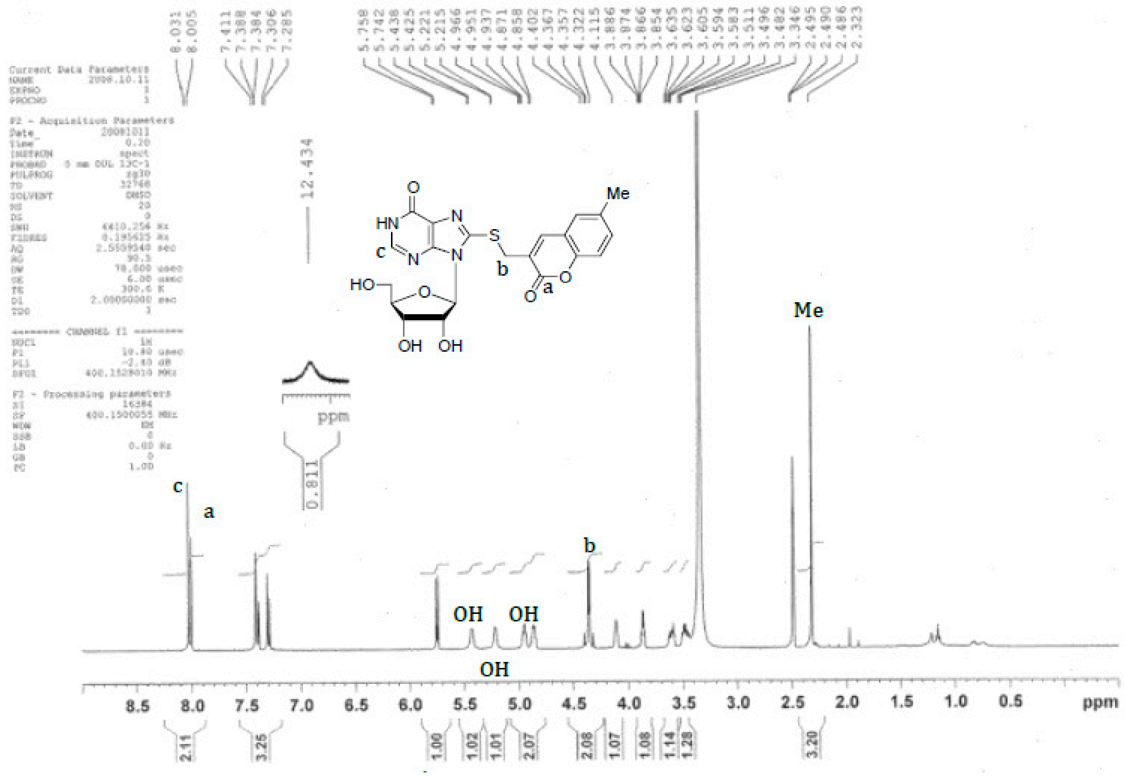

Figure S33. ^{1}H NMR spectrum of compound **9f**.

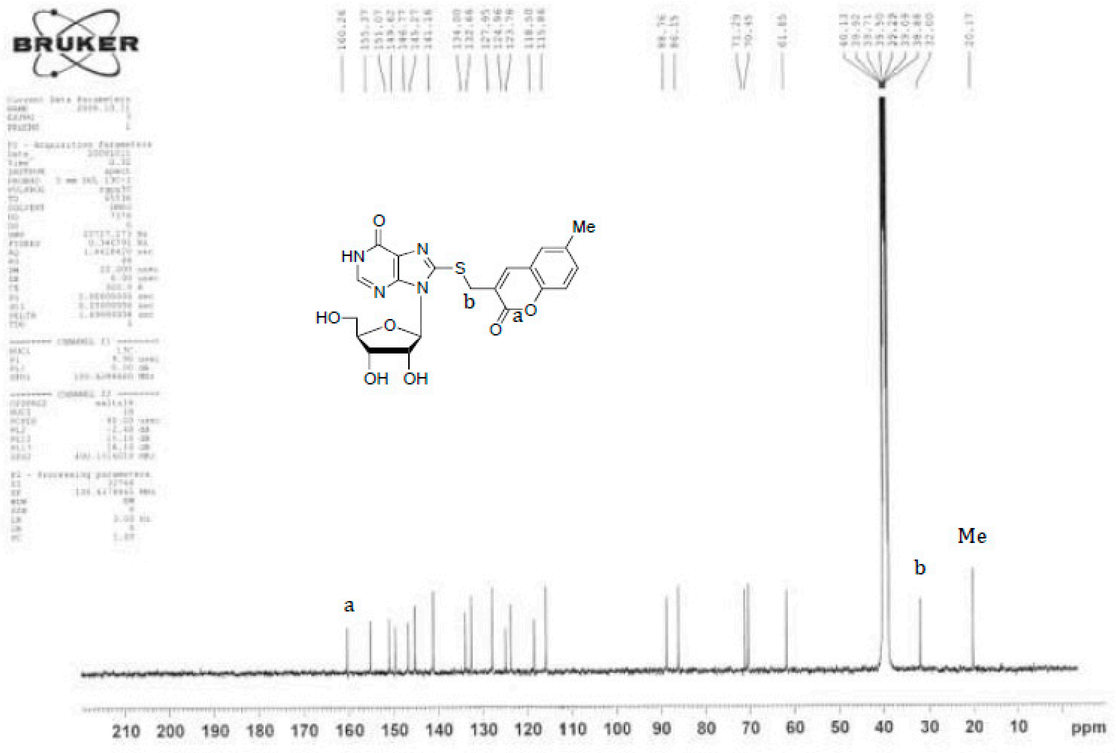

Figure S34. ^{13}C NMR spectrum of compound **9f**.

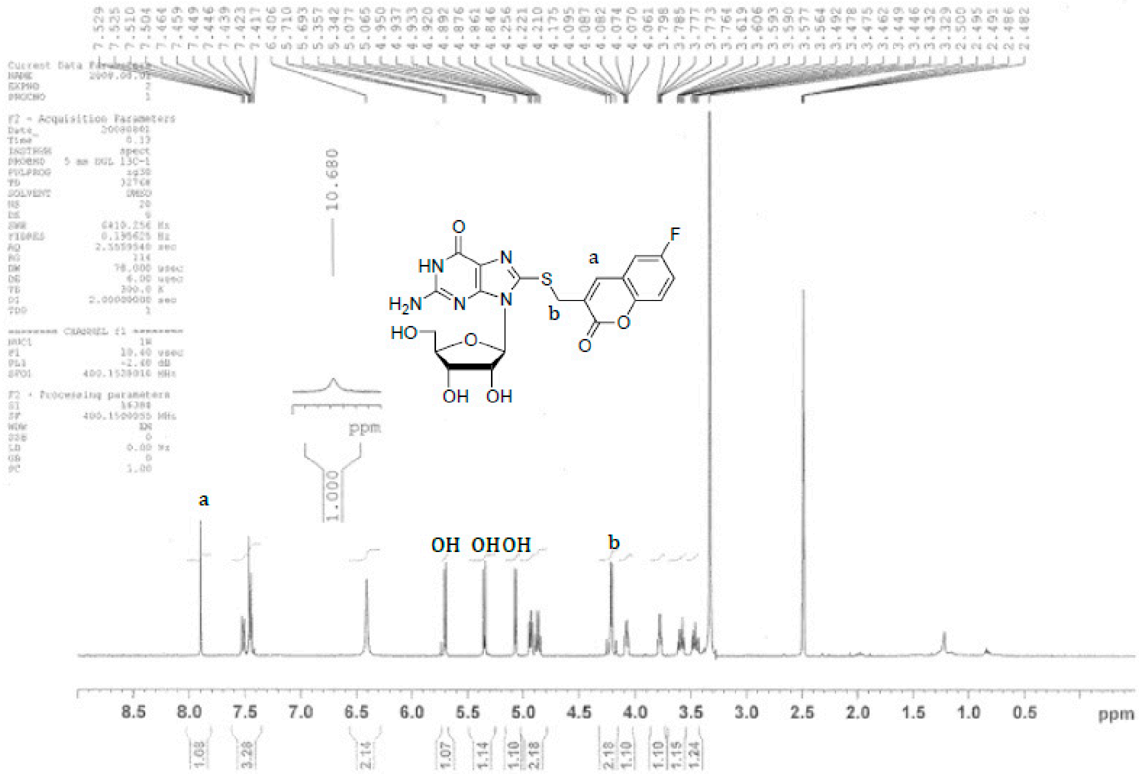

Figure S35. ^{1}H NMR spectrum of compound **11b**.

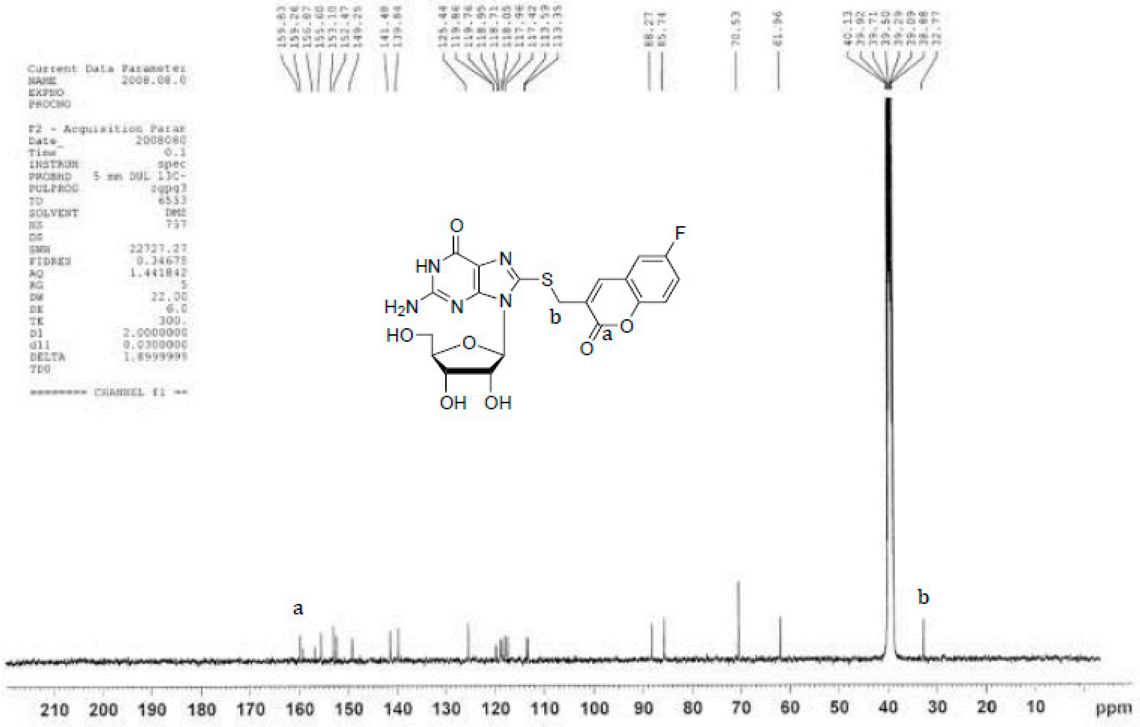

Figure S36. ^{13}C NMR spectrum of compound **11b**.

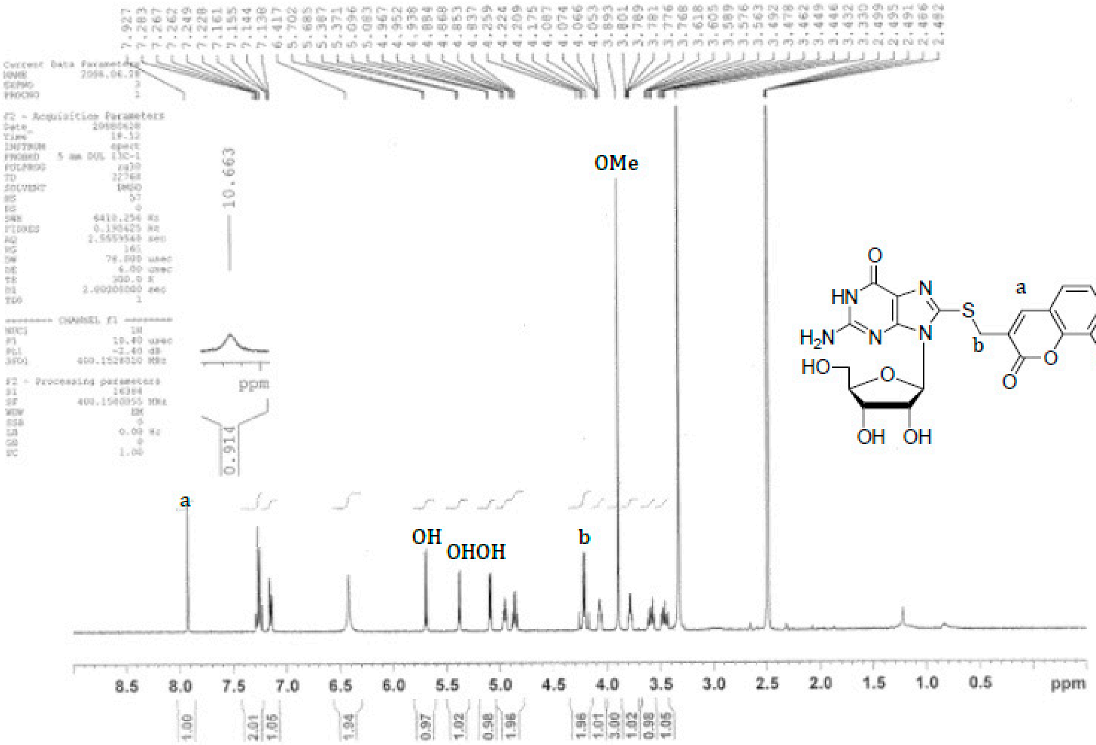

Figure S37. ^{1}H NMR spectrum of compound **11e**.

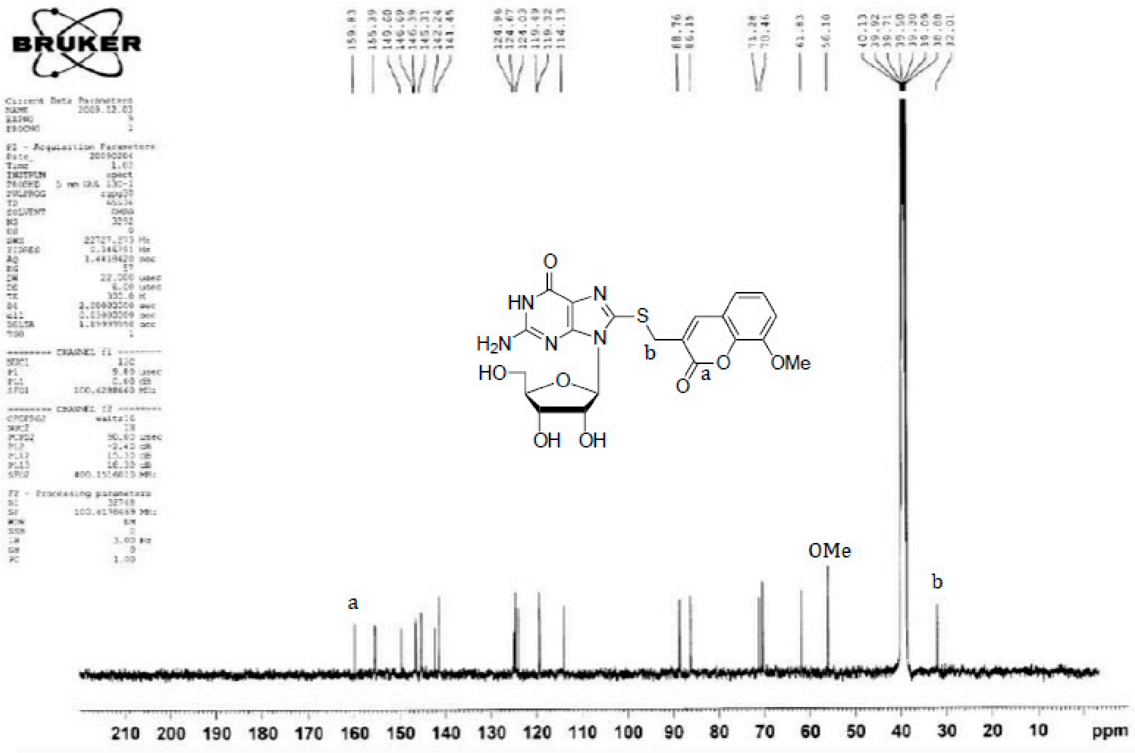

Figure S38. ^{13}C NMR spectrum of compound **11e**.

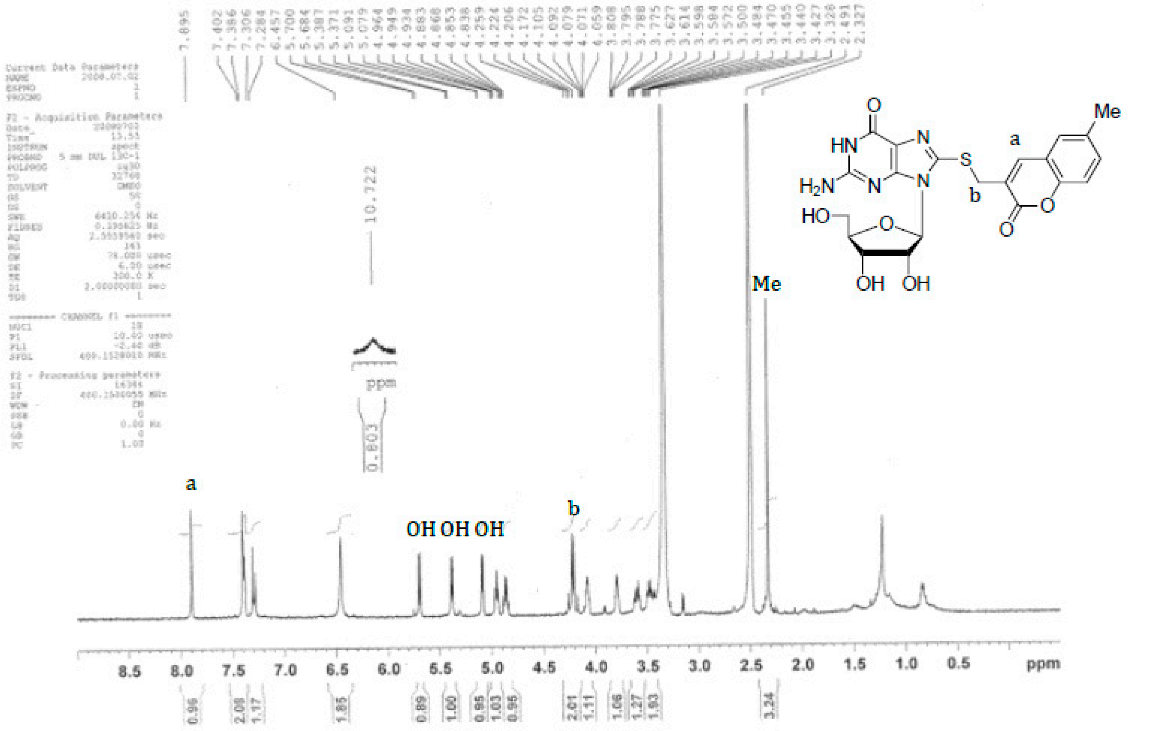

Figure S39. ^{1}H NMR spectrum of compound **11f**.

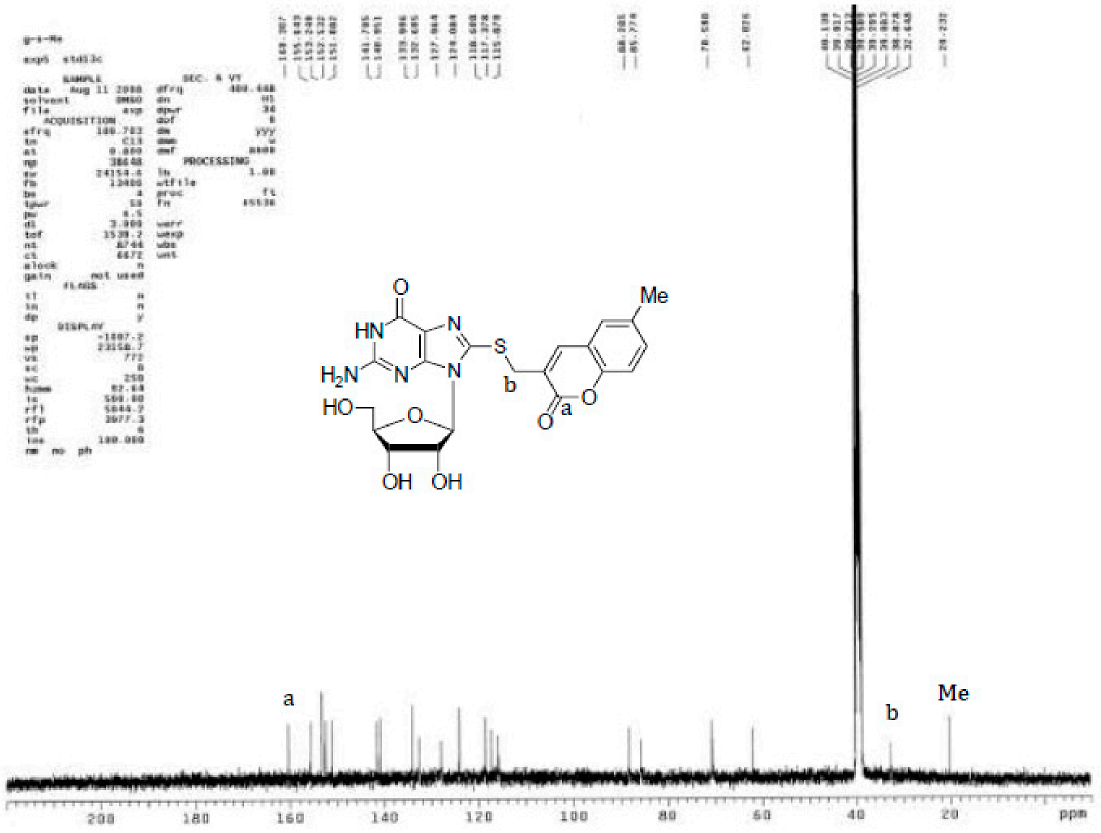

Figure S40. ^{13}C NMR spectrum of compound **11f**.
